# Supplementary material for: Light regulates nuclear detainment of intron-retained transcripts through COP1-spliceosome to modulate photomorphogenesis
Source: Nat Commun. 2024 Jun 15;15:5130. doi: 10.1038/s41467-024-49571-9 (PMC11180117; doi:10.1038/s41467-024-49571-9)
Supplement: Supplementary file 1 — Supplementary Information [file 41467_2024_49571_MOESM1_ESM.pdf]

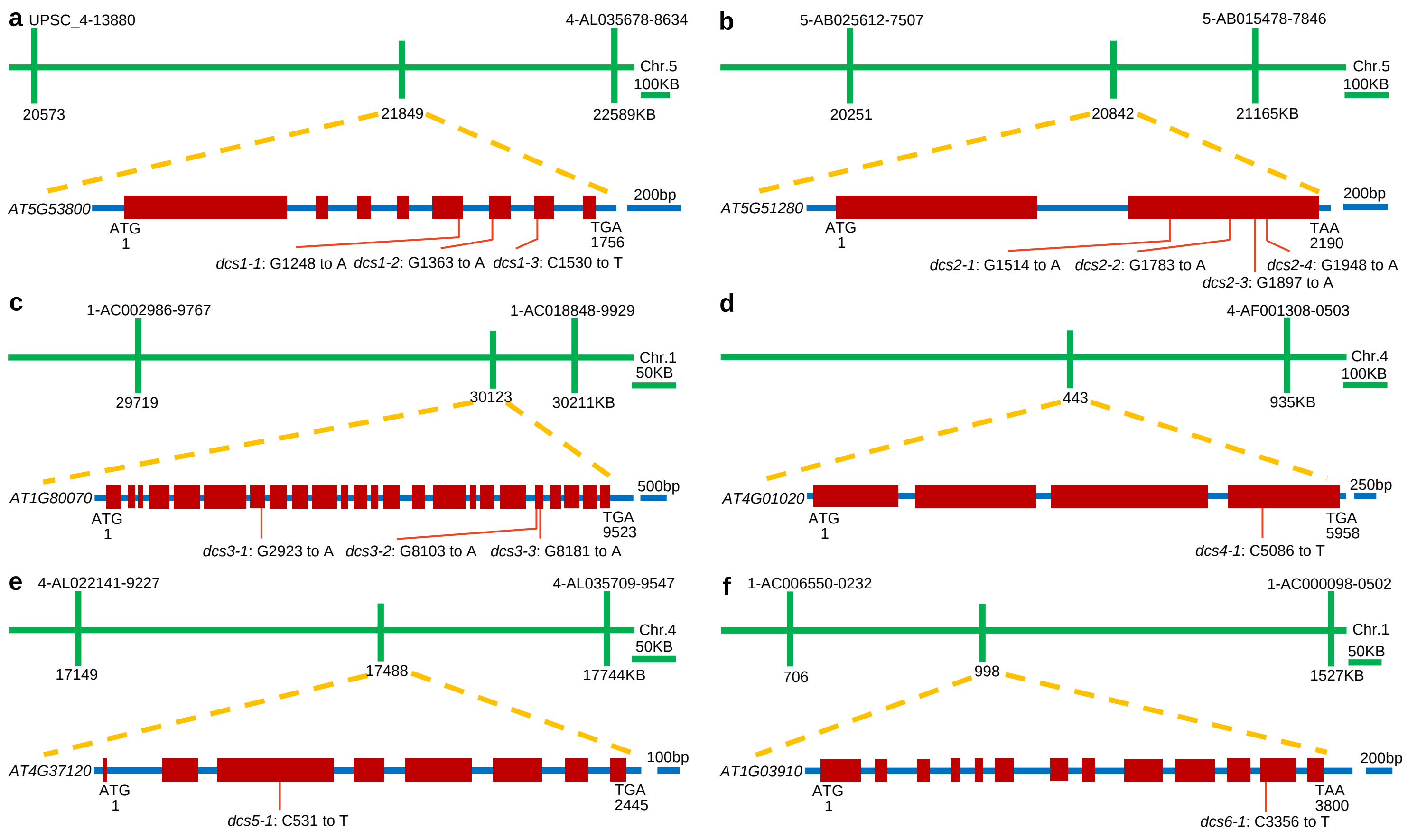

Supplementary Fig. 1. Map-based cloning of *Dominant cop1-6 Suppressor* (*DCS*) genes. Map of the *DCS* genes. Gene loci and structures of *DCS1* (**a**), *DCS2* (**b**), *DCS3* (**c**), *DCS4* (**d**), *DCS5* (**e**), and *DCS6* (**f**) are presented. The exons are represented by boxes, and the introns are represented by lines. The positions and nucleotide changes in each mutant are shown.

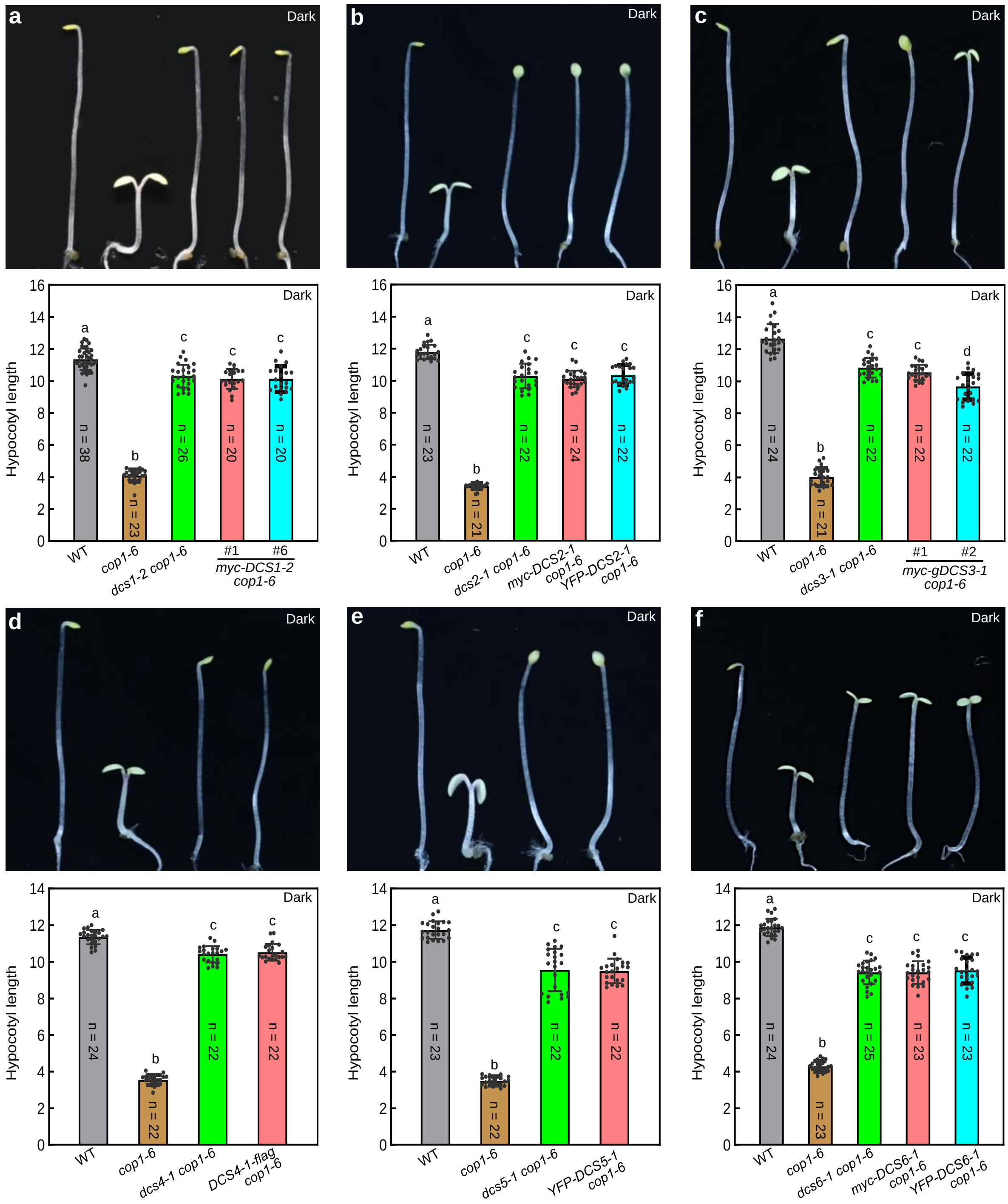

Supplementary Fig. 2. Genomic complementation test. *DCS1-2* (**a**), *DCS2-1* (**b**), *DCS3-1* (**c**), *DCS4-1* (**d**), *DCS5-1* (**e**), and *DCS6-1* (**f**) suppress the constitutive photomorphogenic phenotype of *cop1-6* grown in the dark. Before hypocotyl phenotype investigation, *Arabidopsis* seedlings were grown in the dark for 5 days. Values are mean ± SEM. Letters above the bars indicate significant differences ($P < 0.05$) as determined by one-way ANOVA with Tukey's post hoc analysis. Source data are provided as a Source Data file.

A. Thaliana		*S. cerevisiae*		*D. melanogaster*		*H. sapiens*		Spliceosome compoment
Gene	Protein	Gene	Protein	Gene	Protein	Gene	Protein	
AT5G53800	DCS1							
AT5G51280	DCS2	NP_014287	Dbp2p	NP_524220	abstrakt (abs)	NP_057306	DDX41	Abundant first in C complex, involved in the second transesterification reaction
AT4G33370								
AT1G80070	DCS3/Prp8a	NP_012035	Prp8	NP_610735	Prp8	NP_006436	PRP8	U5 snRNP, involved in the two transesterification reactions
AT4G38780	Prp8b							
AT4G01020	DCS4							
AT5G10370								
AT1G65660	SMP1	NP_010373	Slu7	NP_651659	Slu7	NP_006416	SLU7	Second step factors/C* complex, involved in 3′ splice site selection
AT4G37120	DCS5/SMP2							
AT1G03910	DCS6/CTN			NP_523422	Cactin	NP_067054	CACTIN	Abundant first in C complex, involved in the second transesterification reaction

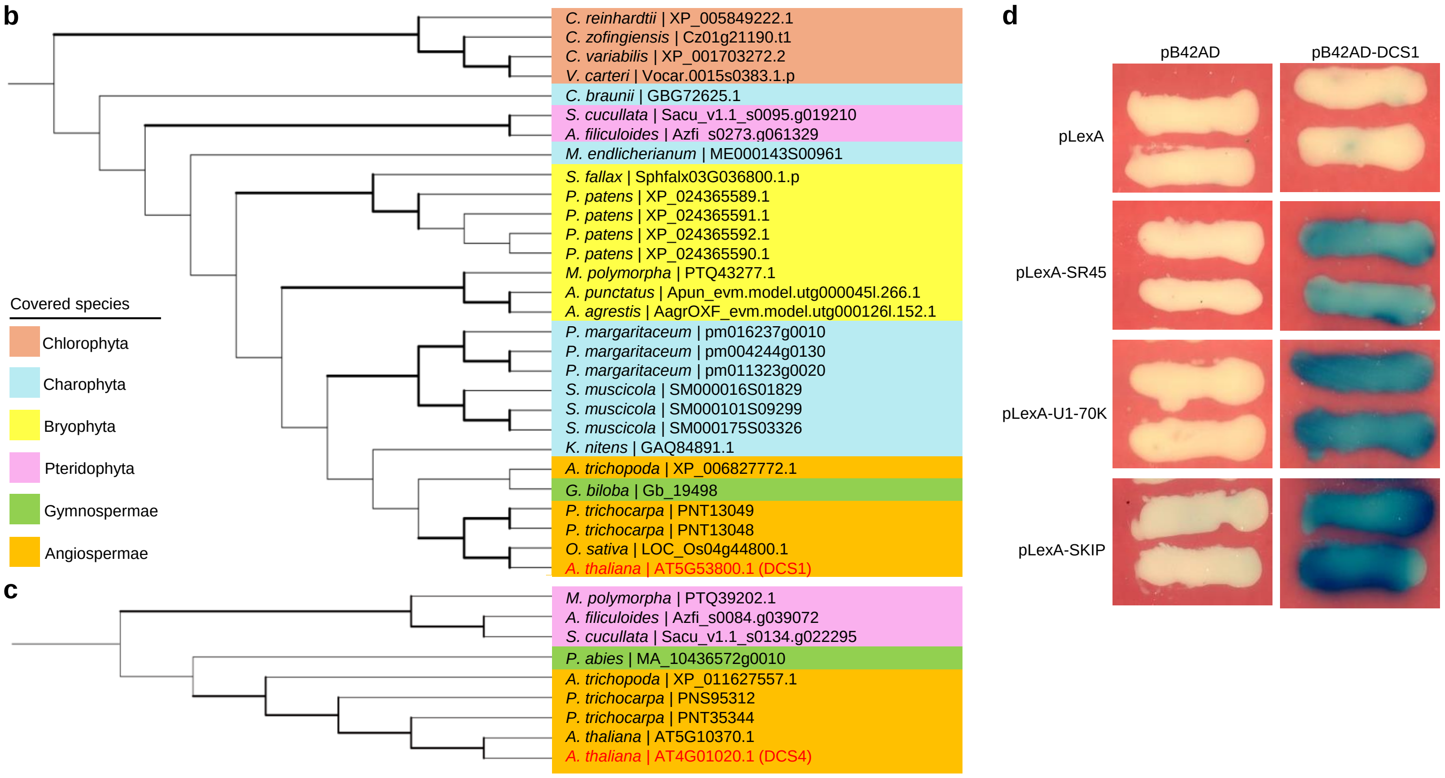

Supplementary Fig. 3. Phylogenetic analyses of DCS proteins. a *Arabidopsis* DCS genes and their homologous genes in yeast, *Drosophila*, and humans. *DCS2*, *DCS3*, *DCS4*, and *DCS5* exist in two copies in the *Arabidopsis* genome. **b, c** Maximum likelihood tree of DCS1 (**b**) and DCS4 (**c**) based on the orthologs identified from genomic data of 31 representative plants, 2 fungi, and 3 animals. The width of the branches indicates a bootstrap value ≥ 50. **d** DCS1 interacts with core spliceosome factors in yeast cells. Yeast two-hybrid assay shows that DCS1 interacts with spliceosome factors SR45, U1-70K, and SKIP.

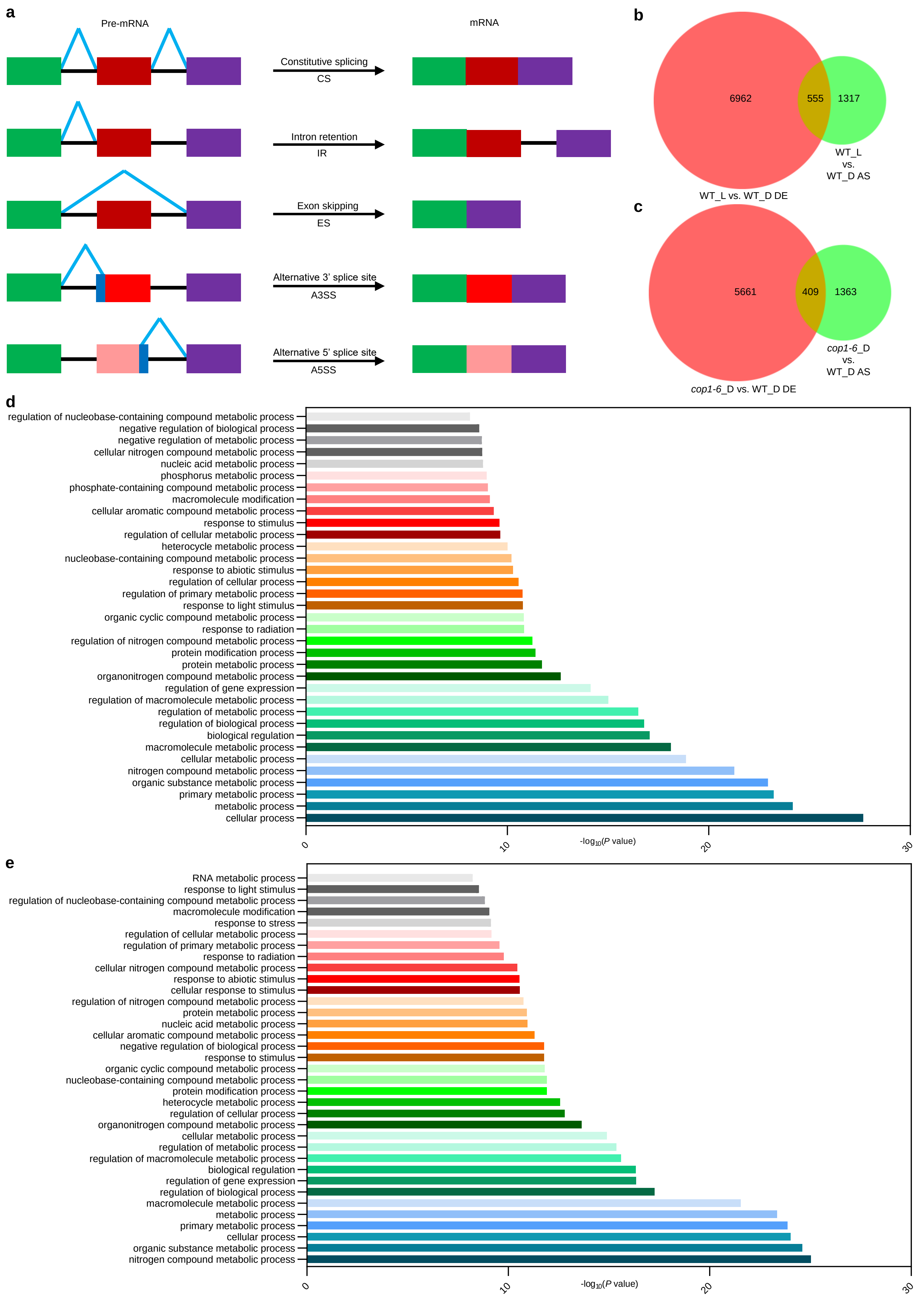

a
Pre-mRNA
mRNA
Constitutive splicing
CS
Intron retention
IR
Exon skipping
ES
Alternative 3' splice site
A3SS
Alternative 5' splice site
A5SS
b
6962
555
1317
WT_L vs. WT_D DE
WT_L vs. WT_D AS
c
5661
409
1363
cop1-6_D vs. WT_D DE
cop1-6_D vs. WT_D AS
d
regulation of nucleobase-containing compound metabolic process
negative regulation of biological process
negative regulation of metabolic process
cellular nitrogen compound metabolic process
nucleic acid metabolic process
phosphorus metabolic process
phosphate-containing compound metabolic process
macromolecule modification
cellular aromatic compound metabolic process
response to stimulus
regulation of cellular metabolic process
heterocycle metabolic process
nucleobase-containing compound metabolic process
response to abiotic stimulus
regulation of cellular process
regulation of primary metabolic process
response to light stimulus
organic cyclic compound metabolic process
response to radiation
regulation of nitrogen compound metabolic process
protein modification process
protein metabolic process
organonitrogen compound metabolic process
regulation of gene expression
regulation of macromolecule metabolic process
regulation of metabolic process
regulation of biological process
biological regulation
macromolecule metabolic process
cellular metabolic process
nitrogen compound metabolic process
organic substance metabolic process
primary metabolic process
metabolic process
cellular process
0
10
20
30
-log10(P value)
e
RNA metabolic process
response to light stimulus
regulation of nucleobase-containing compound metabolic process
macromolecule modification
response to stress
regulation of cellular metabolic process
regulation of primary metabolic process
response to radiation
cellular nitrogen compound metabolic process
response to abiotic stimulus
cellular response to stimulus
regulation of nitrogen compound metabolic process
protein metabolic process
nucleic acid metabolic process
cellular aromatic compound metabolic process
negative regulation of biological process
response to stimulus
organic cyclic compound metabolic process
nucleobase-containing compound metabolic process
protein modification process
heterocycle metabolic process
regulation of cellular process
organonitrogen compound metabolic process
cellular metabolic process
regulation of metabolic process
regulation of macromolecule metabolic process
biological regulation
regulation of gene expression
regulation of biological process
macromolecule metabolic process
metabolic process
primary metabolic process
cellular process
organic substance metabolic process
nitrogen compound metabolic process
0
10
20
30
-log10(P value)

Supplementary Fig. 4. Global analysis of genes regulated by light and COP1. a Modes of the four types of AS in this study, including intron retention (IR), exon skipping (ES), alternative 3' splice site (A3SS), and alternative 5' splice site (A5SS). The exons are represented by boxes, the introns are represented by lines, and splicing events are represented by broken lines (blue). **b, c** Venn diagrams showing the number of genes that displayed light- (**b**) and COP1-dependent (**c**) changes in AS, DE, or in both AS and DE. AS, $P < 0.05$. DE, FC ≥ 2, FDR ≤ 0.05. **d, e** Top 35 enriched GO terms (biological process aspect) for significantly light-dependent (**d**) and COP1-dependent (**e**) AS-changed genes. Source data are provided as a Source Data file.

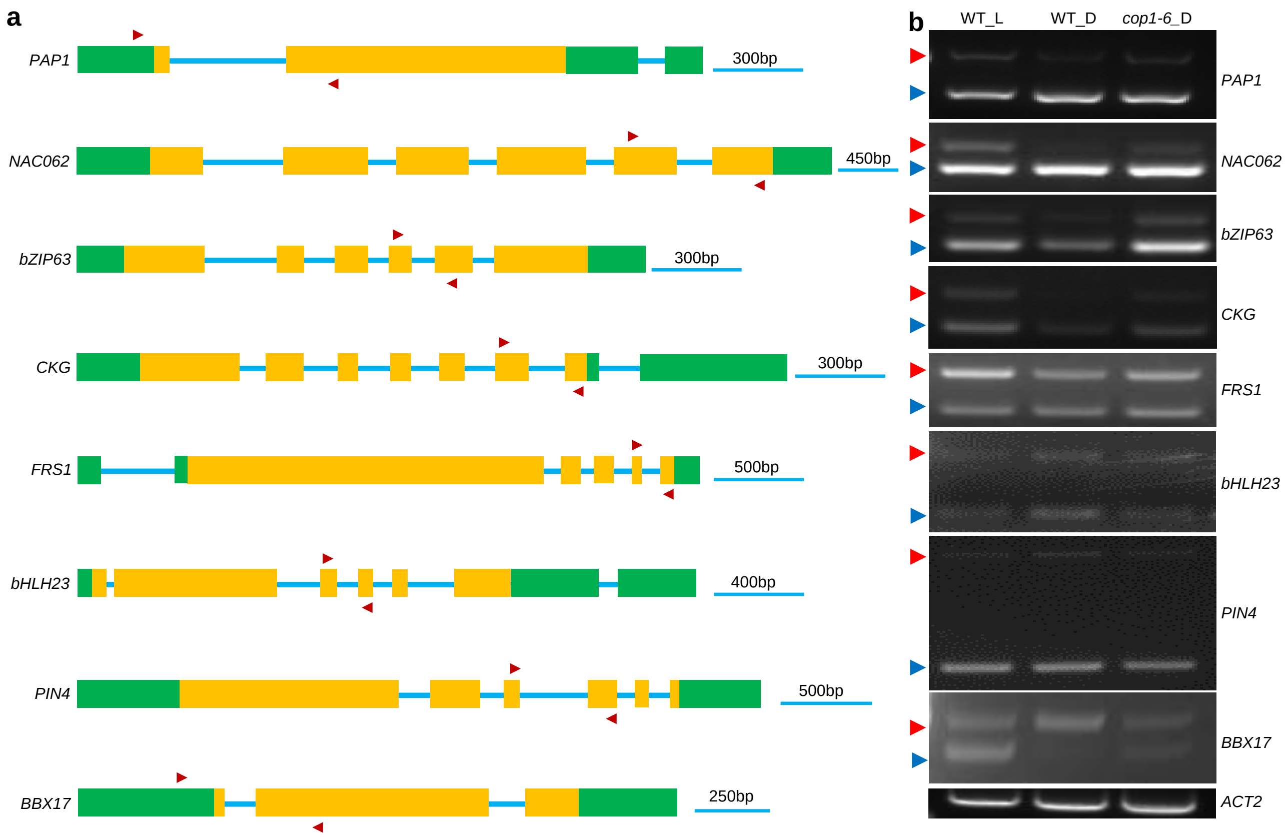

Supplementary Fig. 5. Validation of the IR events identified by mRNA-seq. a Gene structures of *PAP1*, *NAC062*, *bZIP63*, *CKG*, *FRS1*, *bHLH23*, *PIN4*, and *BBX17*. Red arrows indicate the locations of the primers used to confirm the IR events of each gene through semi-quantitative RT-PCR. The exons are represented by yellow boxes, the UTR regions are represented by green boxes, and the introns are represented by blue lines. **b** IR event conformation using semi-quantitative RT-PCR. Red arrows indicate the positions of the IRTs. Blue arrows indicate the positions of the intron-spliced transcripts. The number of PCR cycles used in this analysis were as follows: 25 cycles for *ACT2*, 27 cycles for *PAP1*, *NAC062*, *bZIP63*, and *CKG*, 29 cycles for *FRS1*, and *PIN4*, 32 cycles for *bHLH23*, and 34 cycles for *BBX17*.

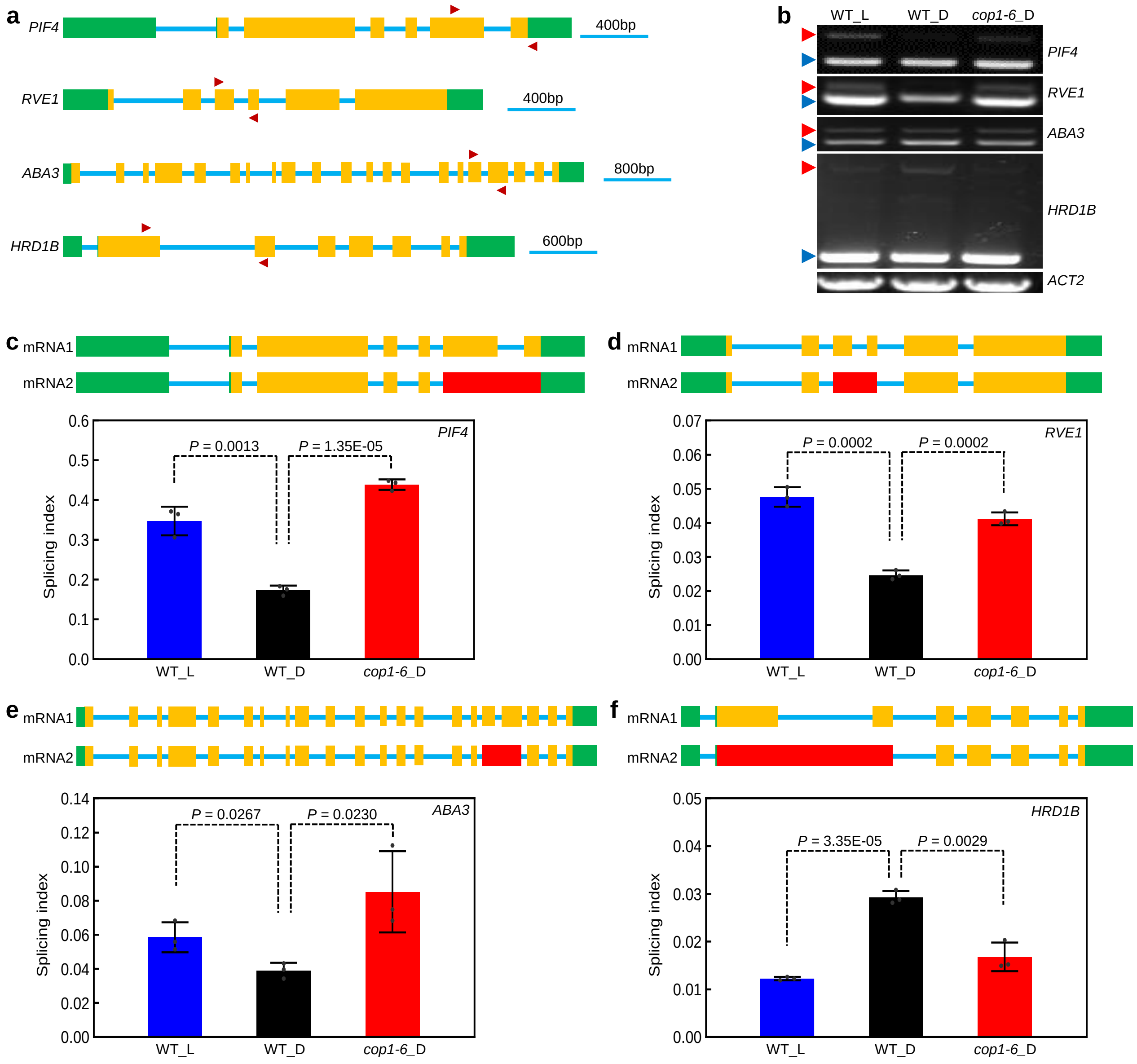

Supplementary Fig. 6. Light and COP1 induce changes in the IRTs of *PIF4*, *RVE1*, *ABA3*, and *HRD1B*. a Gene structures of *PIF4*, *RVE1*, *ABA3*, and *HRD1B*. Red arrows indicate the locations of the primers used to confirm the IR events of each gene through semi-quantitative RT-PCR. The exons are represented by yellow boxes, the UTR regions are represented by green boxes, and the introns are represented by blue lines. **b** IR event conformation using semi-quantitative RT-PCR. Red arrows indicate the positions of the IRTs. Blue arrows indicate the positions of the intron-spliced transcripts. The number of PCR cycles used in this analysis were as follows: 25 cycles for *ACT2*, 28 cycles for *PIF4*, *RVE1*, and *ABA3*, and 31 cycles for *HRD1B*. **c–f** Light and COP1 induce changes in the SI of *PIF4* (**c**), *RVE1* (**d**), *ABA3* (**e**), and *HRD1B* (**f**). The exons are represented by yellow boxes, the UTR regions are represented by green boxes, and the introns are represented by blue lines. The red boxes represent the intron-retaining exons. mRNA-1 represents the splice variant that is considered to encode the functional full-length protein, while mRNA-2 represents the intron-containing splice variant identified by mRNA-seq. The splicing index, defined as the abundance of mRNA2 relative to total mRNA level, was checked using qRT-PCR. Values are mean $\pm$ SEM (n = 3). Significance is evaluated by the two-sided Student's *t*-test and *P* values are indicated above the bars. Source data are provided as a Source Data file.

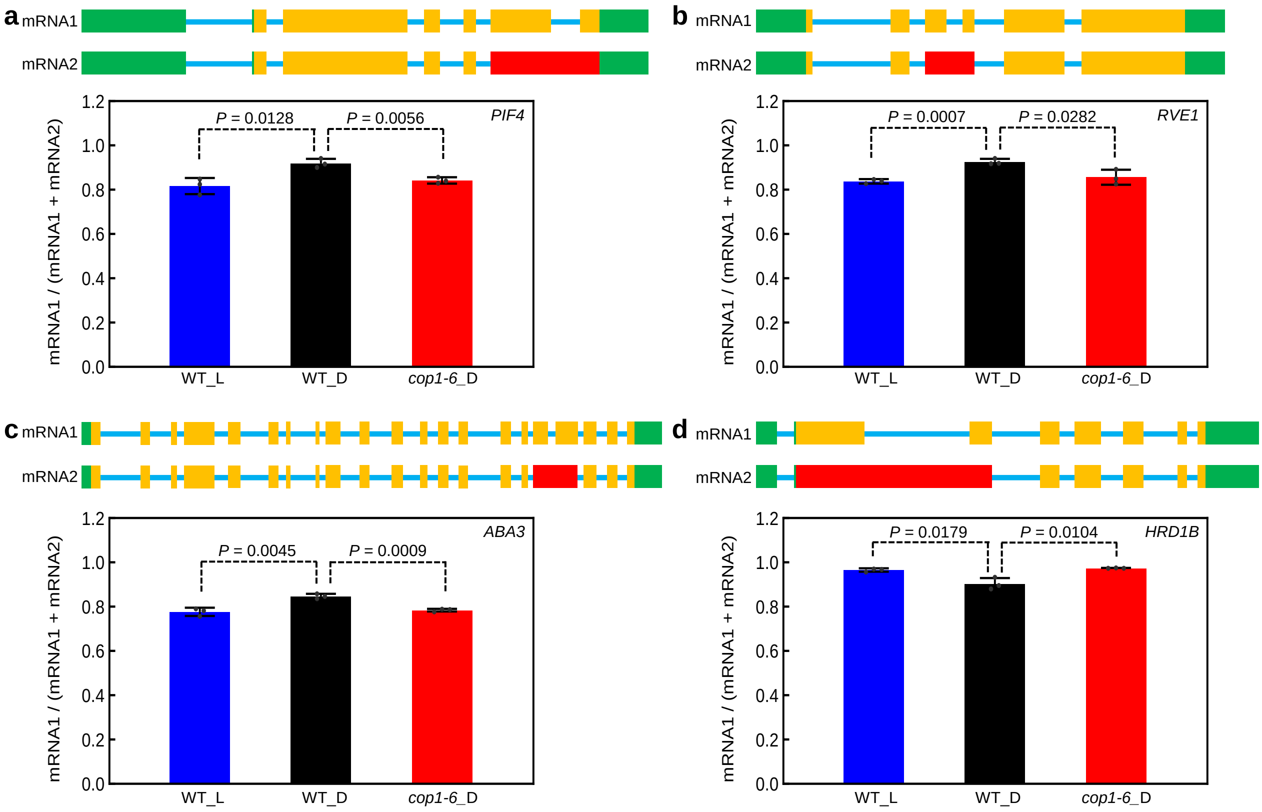

Supplementary Fig. 7. Light and COP1 induce changes in the functional transcripts of *PIF4* (a), *RVE1* (b), *ABA3* (c), and *HRD1B* (d). Total RNA was extracted from 5-d-old WT_L, WT_D, and *cop1-6*_D seedlings. The exons are represented by yellow boxes, the UTR regions are represented by green boxes, and the introns are represented by blue lines. The red boxes represent the intron-retaining exons. mRNA-1 represents the splice variant considered to encode the functional full-length protein, while mRNA-2 represents the intron-containing splice variant identified by mRNA-seq. The values are shown as the mean ± SEM (n = 3). Significance is evaluated by the two-sided Student's *t*-test and *P* values are indicated above the bars. Source data are provided as a Source Data file.

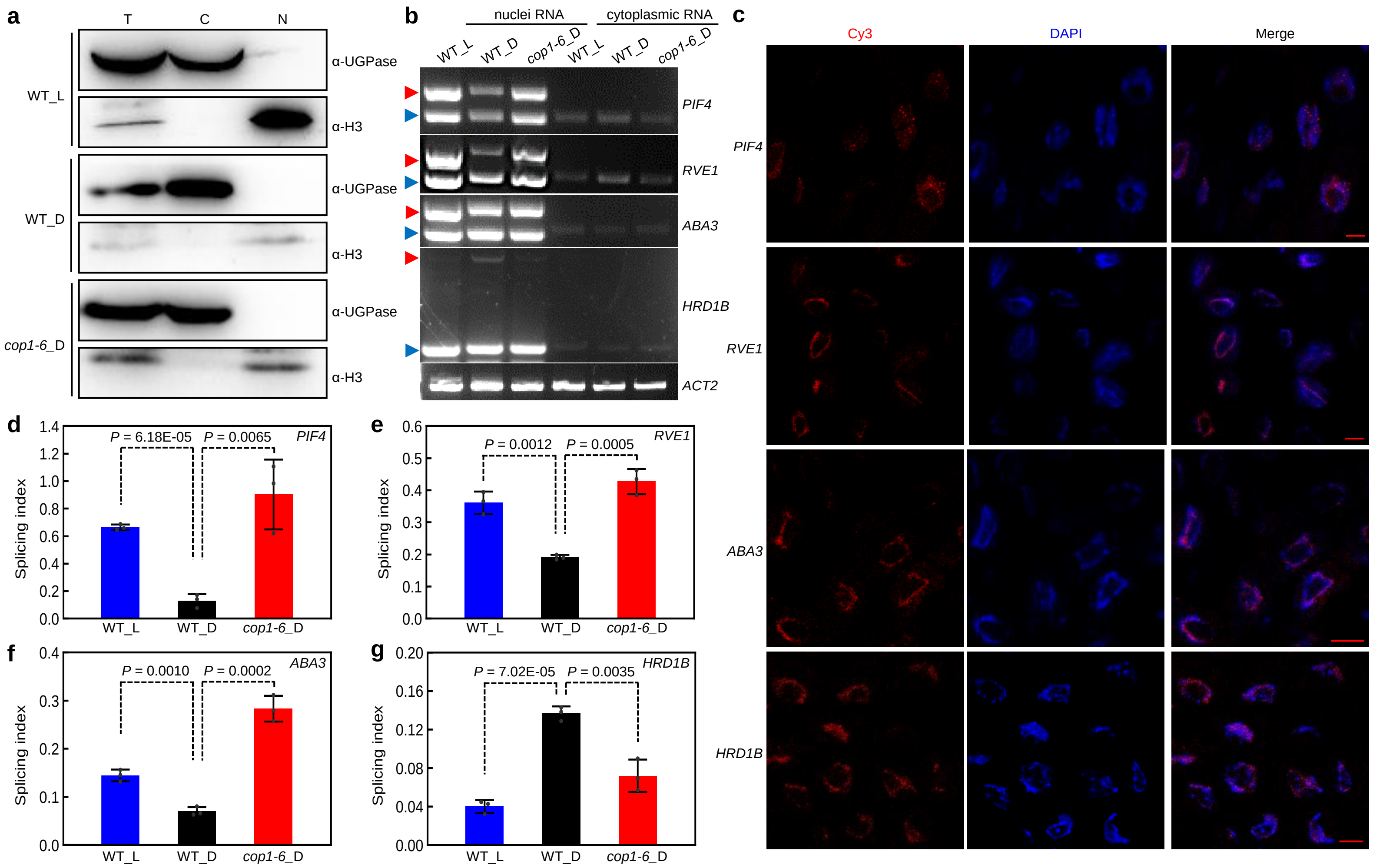

Supplementary Fig. 8. Intron-retained transcripts localize in the nucleus. a Separation of nuclear and cytoplasmic fractions. Western blot analysis showing the distribution in the subcellular fractions of UGPase, a cytosolic marker protein, and Histone 3 (H3), a nuclear marker protein. **b** Changes in the abundance of alternative splicing isoforms in the nuclear and cytoplasmic fractions of WT_L, WT_D, and *cop1-6*_D. The IRTs of *PIF4*, *RVE1*, *ABA3*, and *HRD1B* are highly enriched in the nucleus, while they are undetectable in the cytosolic fraction. Red arrows indicate the positions of the IRTs. Blue arrows indicate the positions of the intron-spliced transcripts. The number of PCR cycles used in this analysis were as follows: 25 cycles for *ACT2*, 29 cycles for *PIF4*, *RVE1*, and *ABA3*, and 32 cycles for *HRD1B*. **c** Subcellular distribution of the IRTs of *PIF4*, *RVE1*, *ABA3*, and *HRD1B*. RNA FISH images showing IRTs of *PIF4*, *RVE1*, *ABA3*, and *HRD1B* in WT seedlings grown under white light for 1 week. *Arabidopsis* WT seedlings were subjected to 5'-Cy3-labeled probes in situ hybridization and DAPI staining, followed by fluorescence microscopy. The 5'-Cy3-labeled probes specifically target the retained introns of *PIF4*, *RVE1*, *ABA3*, and *HRD1B*. Merge, merged images of the cy3 channel and the DAPI channel. Bar, 10 µm. **d–g** Light- and COP1-induced changes in the SI values of *PIF4* (**d**), *RVE1* (**e**), *ABA3* (**f**), and *HRD1B* (**g**) in the nucleus. The values are shown as the mean ± SEM (n = 3). Significance is evaluated by the two-sided Student's *t*-test and *P* values are indicated above the bars. Source data are provided as a Source Data file.

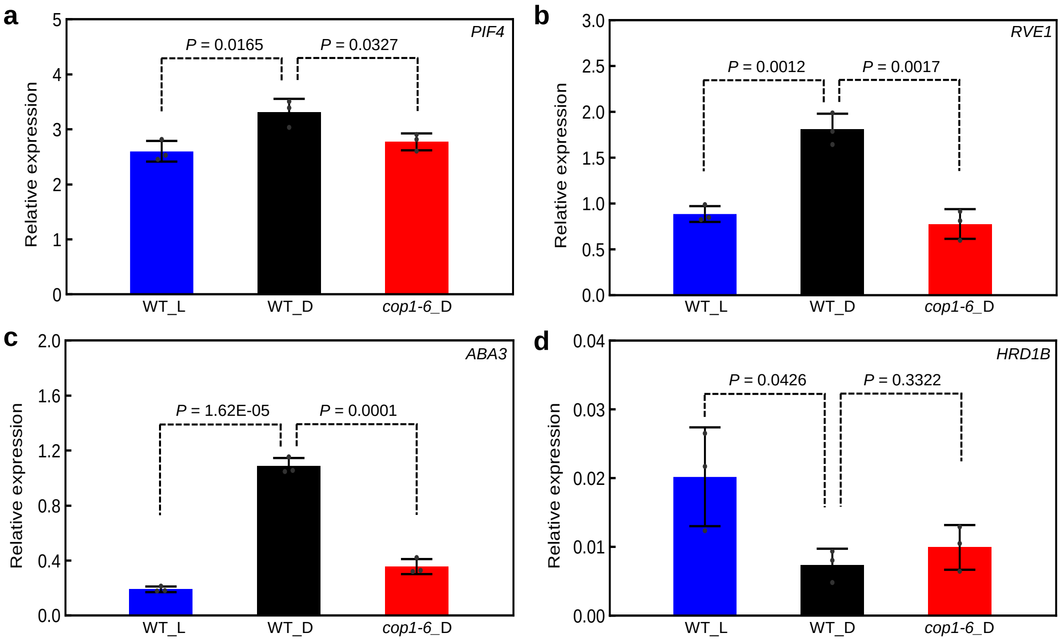

Supplementary Fig. 9. The expression of *PIF4* (a), *RVE1* (b), *ABA3* (c), and *HRD1B* (d) in the cytoplasmic fraction of WT_L, WT_D, and *cop1-6*_D seedlings. Cytoplasmic RNA was extracted from 5-d-old WT_L, WT_D, and *cop1-6*_D seedlings. The values are shown as the mean ± SEM (n = 3). Significance is evaluated by the two-sided Student's *t*-test and *P* values are indicated above the bars. Source data are provided as a Source Data file.

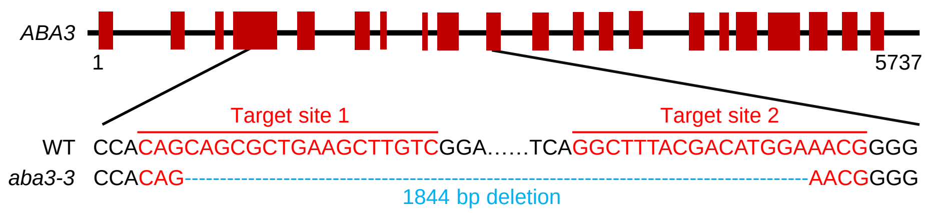

Supplementary Fig. 10. The DNA sequence of *aba3-3* mutant created by the CRISPER/Cas9 system. The exons are represented by red boxes, and the introns are represented by lines. '-' in blue indicates nucleic acid deletion.

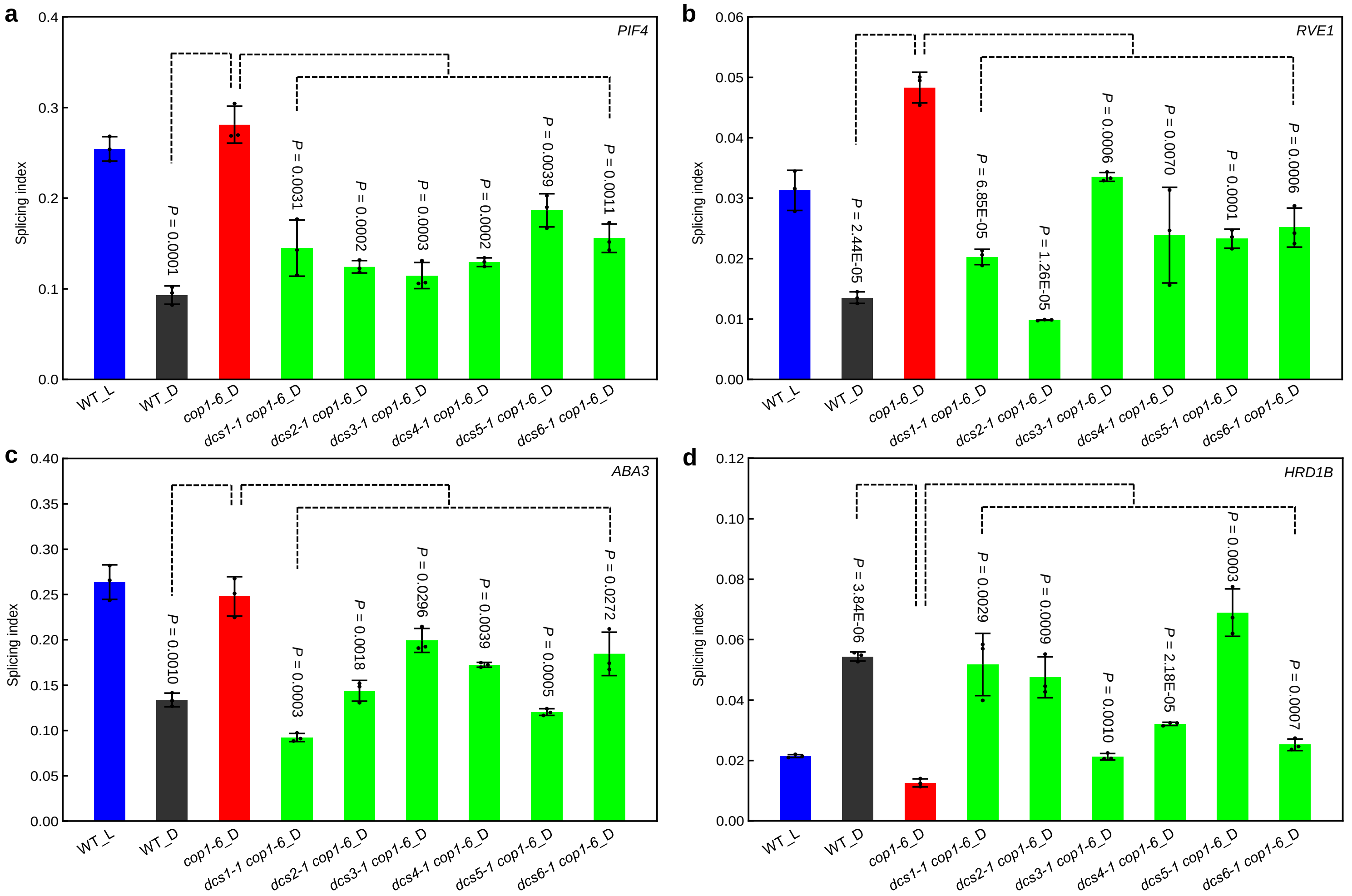

Supplementary Fig. 11. The *dcs* suppressors partially rescue the SI of *PIF4*, *RVE1*, *ABA3*, and *HRD1B* in the *cop1-6* mutant. SI of *PIF4* (**a**), *RVE1* (**b**), *ABA3* (**c**), and *HRD1B* (**d**) in WT_L, WT_D, *cop1-6*_D, and dark-grown *dcs cop1-6* double mutants. The values are shown as the mean ± SEM (n = 3). Significance is evaluated by the two-sided Student's *t*-test and *P* values are indicated above the bars. Source data are provided as a Source Data file.

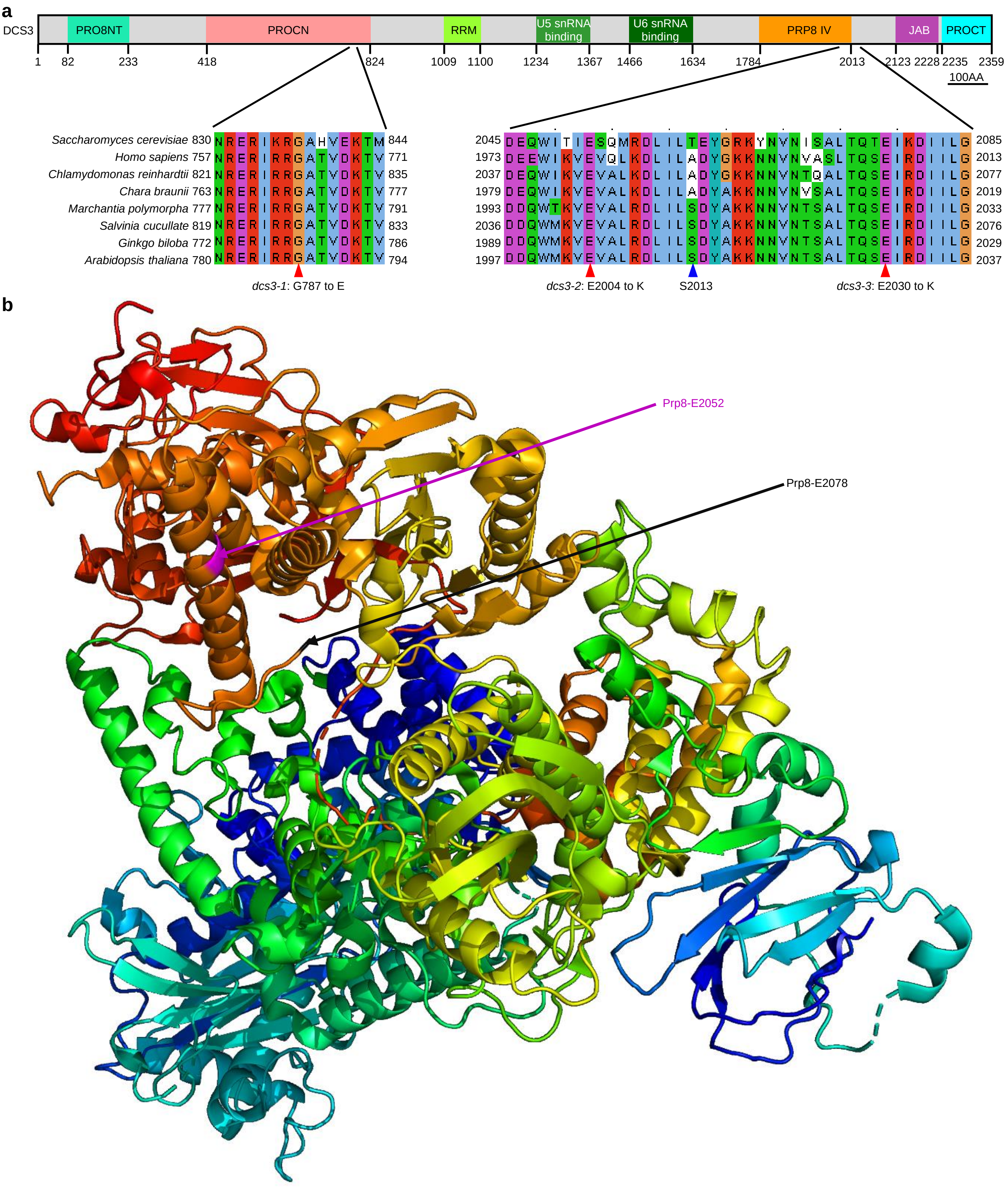

Supplementary Fig. 12. E2004 and E2030 of DCS3 might localize on the surface of DCS3. a Sequence alignment of the conserved DCS3 regions containing G787E, E2004K, and E2030K mutations in *dcs3-1*, *dcs3-2*, and *dcs3-3*, respectively. The point mutation sites in *dcs3* mutants are labeled by red triangles, while the blue triangle indicates the position of the right border of the PRP8 IV domain. **b** Crystal structure of the Prp8–Aar2 complex (PDB accession 4I43). The positions of Prp8-E2052 and Prp8-E2078 are colored magenta and black, respectively, in the Prp8 protein structure. Both Prp8-E2052 and Prp8-E2078 are highly conserved among species. Prp8-E2052 and Prp8-E2078 refer to DCS3-E2004 and DCS3-E2030, respectively, in *Arabidopsis*. The protein structure is displayed using PyMOL v2.5 (Schrödinger, LLC).

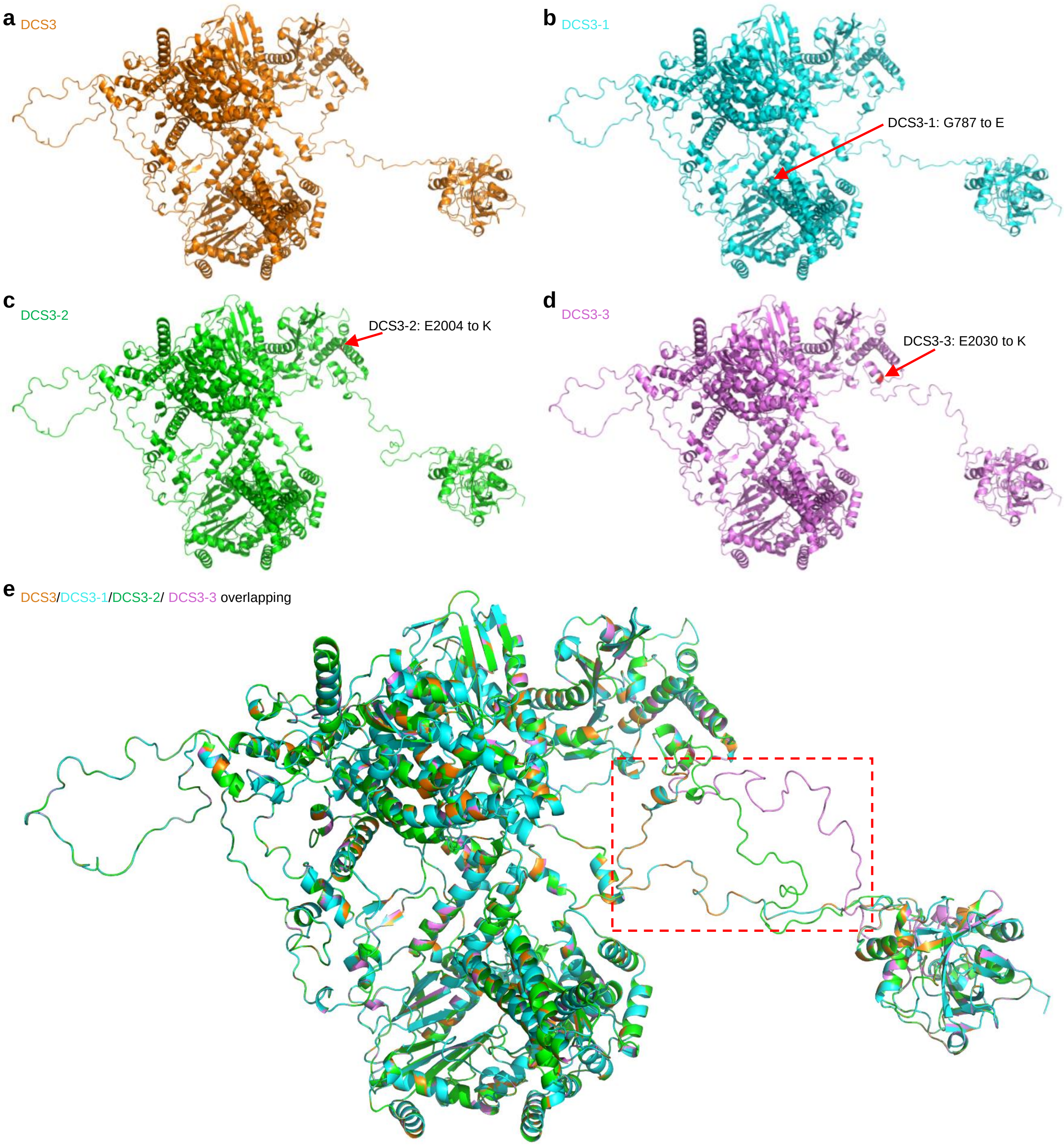

Supplementary Fig. 13. The point mutations in *dcs3-2* and *dcs3-3* change the protein structure of DCS3. a-**d** The protein structures of DCS3 (**a**), DCS3-1 (**b**), DCS3-2 (**c**), and DCS3-3 (**d**) predicted by SWISS-MODEL using the reported human Prp8 structure (SMTL ID: 5yzg.1) as template. **e** Structural alignment of DCS3, DCS3-1, DCS3-2, and DCS3-3 proteins using PyMOL v2.5 (Schrödinger, LLC). The protein structure changes induced by the point mutations in *dcs3-2* and *dcs3-3* are marked in red box.

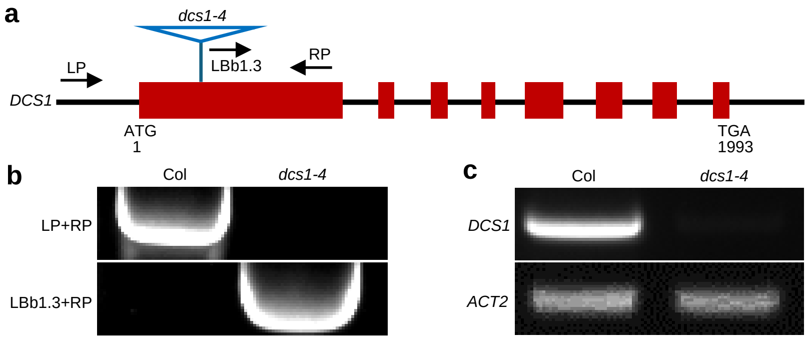

Supplementary Fig. 14. Identification and characterization of *dcs1-4* mutant. a The T-DNA insertion position in the *dcs1-4* mutant. **b** Identification of *dcs1-4* by PCR analysis. LP, left primer. RP, right primer. LBb1.3, T-DNA left border primer. **c** Semi-quantitative RT-PCR analysis of *DCS1* expression in WT and *dcs1-4*. The number of PCR cycles used in this analysis were as follows: 25 cycles for *ACT2*, and 30 cycles for *DCS1*.

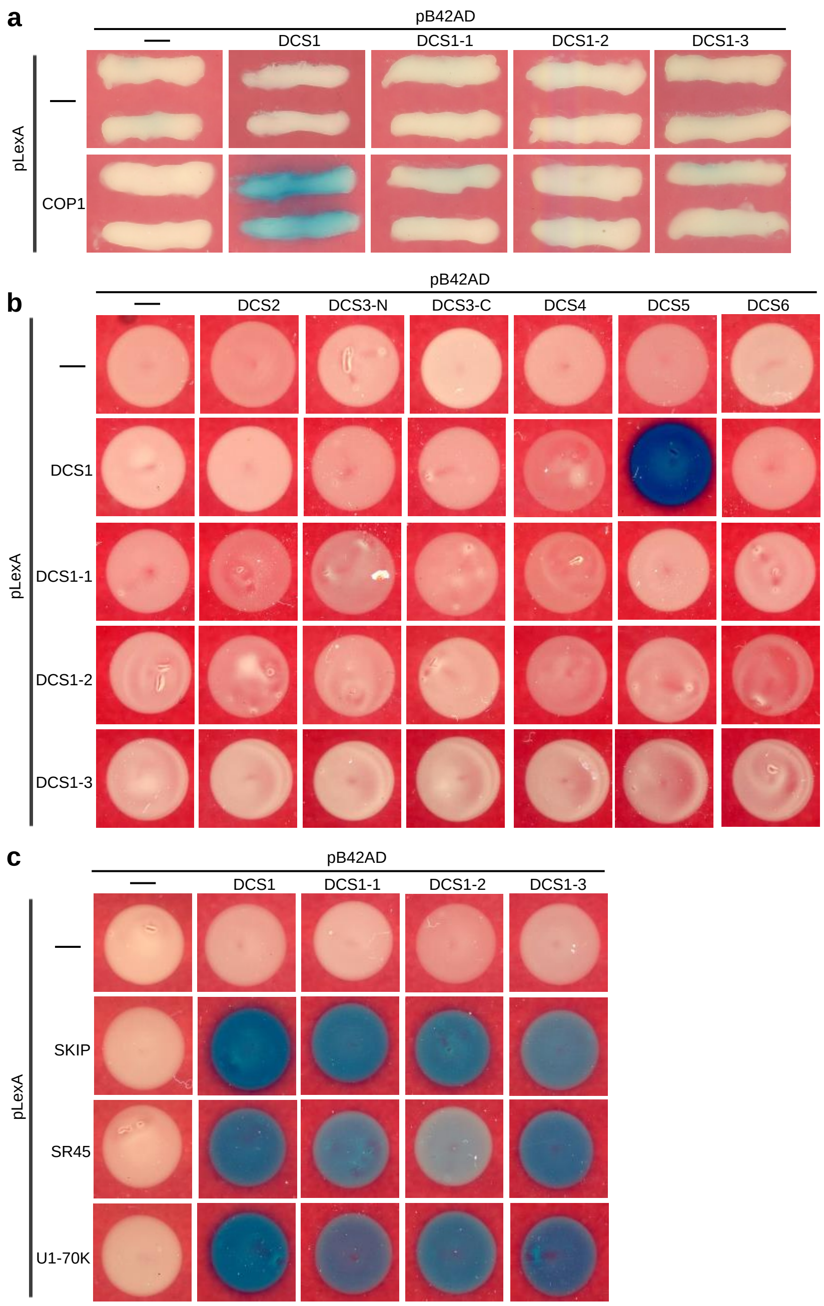

Supplementary Fig. 15. The mutations in DCS1 affect protein–protein interactions. a Yeast-two-hybrid screening for the interactions between DCS1 proteins and COP1. **b** Yeast two-hybrid screening for the interactions between DCS1 proteins and DCS2, DCS3-N, DCS3-C, DCS5, and DCS6. **c** Yeast two-hybrid screening for the interactions between DCS1 proteins and the splicing factors SKIP, SR45, and U1-70K.

Supplementary Table 1. Reference genomes, related to Supplementary Fig. 3.

Category	Species	Source
Rhodophyta	*Cyanidioschyzon merolae*	https://ftp.ncbi.nlm.nih.gov/genomes/all/GCF/000/091/205/GCF_000091205.1_ASM9120v1/
	Galdieria sulphuraria	http://plants.ensembl.org/Galdieria_sulphuraria/Info/Index
	Porphyridium purpureum	https://ftp.ncbi.nlm.nih.gov/genomes/all/GCA/008/690/995/GCA_008690995.1_P_purpureum_CCMP1328_Hybrid_assembly/
	Chondrus crispus	http://plants.ensembl.org/Chondrus_crispus/Info/Index
Chlorophyta	*Ostreococcus tauri*	https://ftp.ncbi.nlm.nih.gov/genomes/all/GCF/000/214/015/GCF_000214015.3_version_140606
	Micromonas pusilla	https://ftp.ncbi.nlm.nih.gov/genomes/all/GCF/000/151/265/GCF_000151265.2_Micromonas_pusilla_CCMP1545_v2.0
	Chlamydomonas reinhardtii	https://ftp.ncbi.nlm.nih.gov/genomes/all/GCF/000/002/595/GCF_000002595.2_Chlamydomonas_reinhardtii_v5.5
	Volvox carteri	https://phytozome.jgi.doe.gov/pz/portal.html
	Chromochloris zofingiensis	https://phytozome.jgi.doe.gov/pz/portal.html
	Chlorella variabilis	https://ftp.ncbi.nlm.nih.gov/genomes/all/GCF/000/147/415/GCF_000147415.1_v_1.0
Charophyta	*Chara braunii*	https://ftp.ncbi.nlm.nih.gov/genomes/all/GCA/003/427/395/GCA_003427395.1_Cbr_1.0
	Chlorokybus atmophyticus	http://ftp.cngb.org/pub/CNSA/data1/CNP0000228/CNS0021447/CNA0002353/
	Klebsormidium nitens	https://ftp.ncbi.nlm.nih.gov/genomes/all/GCA/000/708/835/GCA_000708835.1_ASM70883v1
	Mesostigma viride	http://ftp.cngb.org/pub/CNSA/data1/CNP0000228/CNS0021438/CNA0002352/
	Spirogloea muscicola	https://figshare.com/articles/dataset/Genomes_of_subaerial_Zygnematophyceae_provide_insights_into_land_plant_evolution/9911876
	Penium margaritaceum	http://bioinfo.bti.cornell.edu/cgi-bin/Penium/home.cgi
	Mesotaenium endlicherianum	https://figshare.com/articles/dataset/Genomes_of_subaerial_Zygnematophyceae_provide_insights_into_land_plant_evolution/9911876
Bryophyta	*Anthoceros agrestis*	https://www.hornworts.uzh.ch/en.html
	Anthoceros punctatus	https://www.hornworts.uzh.ch/en.html
	Physcomitrium patens	https://ftp.ncbi.nlm.nih.gov/genomes/all/GCF/000/002/425/GCF_000002425.4_Phypa_V3
	Sphagnum fallax	https://phytozome-next.jgi.doe.gov/info/Sfallax_v1_1
	Marchanta polymorpha	https://ftp.ncbi.nlm.nih.gov/genomes/all/GCA/003/032/435/GCA_003032435.1_Marchanta_polymorpha_v1
Pteridophyta	*Selaginella moellendorffii*	https://phytozome-next.jgi.doe.gov/info/Smoellendorffii_v1_0
	Azolla filiculoides	https://www.fernbase.org/ftp/Azolla_filiculoides/Azolla_asm_v1.1/Azolla_filiculoides.protein.highconfidence_v1.1.fasta
	Salvinia cucullata	https://www.fernbase.org/ftp/Salvinia_cucullata/Salvinia_asm_v1.2/Salvinia_cucullata.protein.highconfidence_v1.2.fasta
Gymnospermae	*Ginkgo biloba*	http://gigadb.org/dataset/view/id/100209
	Picea abies	ftp://plantgenie.org/Data/ConGenIE/Picea_abies/v1.0/FASTA/GenePrediction/Pabies1.0-all-pep.faa.gz
Angiospermae	*Amborella trichopoda*	https://ftp.ncbi.nlm.nih.gov/genomes/all/GCF/000/471/905/GCF_000471905.2_AMTR1.0
	Arabidopsis thaliana	http://plants.ensembl.org/Arabidopsis_thaliana/Info/Index
	Oryza sativa	https://ftp.ncbi.nlm.nih.gov/genomes/all/GCF/001/433/935/GCF_001433935.1_IRGSP-1.0/GCF_001433935.1_IRGSP-1.0_protein.faa.gz
	Populus trichocarpa	http://ftp.ensemblgenomes.org/pub/plants/release-52/fasta/populus_trichocarpa/pep/Populus_trichocarpa.Pop_tri_v3.pep.all.fa.gz
Fungi	*Saccharomyces cerevisiae*	https://ftp.ncbi.nlm.nih.gov/genomes/all/GCF/000/146/045/GCF_000146045.2_R64/GCF_000146045.2_R64_protein.faa.gz
	Neurospora crassa	https://ftp.ncbi.nlm.nih.gov/genomes/all/GCF/000/182/925/GCF_000182925.2_NC12/GCF_000182925.2_NC12_protein.faa.gz
Animal	*Drosophila melanogaster*	http://ftp.ensemblgenomes.org/pub/metazoa/release-53/fasta/drosophila_melanogaster/pep/Drosophila_melanogaster.BDGP6.32.pep.all.fa.gz
	Mus musculus	http://ftp.ensembl.org/pub/release-106/fasta/mus_musculus/pep/Mus_musculus.GRCm39.pep.all.fa.gz
	Homo sapiens	http://ftp.ensembl.org/pub/release-106/fasta/homo_sapiens/pep/Homo_sapiens.GRCh38.pep.all.fa.gz

Supplementary Table 2. Mass spectrometry data analysis, related to Fig. 6.
Genes in this list are according to Shikata et al. (2014).

Gene Name	GeneID	Group	YFP-DCS1 IP	YFP-DCS2 IP	HA-DCS3 IP	FLAG-DCS4 IP	YFP-DCS5 IP	YFP-DCS6 IP
atSmB-a	AT5G44500	Sm core Proteins	0	0	0	0	0	0
atSmB-b	AT4G20440	Sm core Proteins	4	1	5	0	2	2
atSmD1-a	AT3G07590	Sm core Proteins	0	0	0	0	0	0
atSmD1-b	AT4G02840	Sm core Proteins	1	0	5	0	1	1
atSmD2-a	AT2G47640	Sm core Proteins	0	0	0	0	0	0
atSmD2-b	AT3G62840	Sm core Proteins	1	0	1	0	0	1
atSmD3-a	AT1G76300	Sm core Proteins	0	0	4	0	0	2
atSmD3-b	AT1G20580	Sm core Proteins	0	2	4	0	0	2
atSmE-a	AT4G30330	Sm core Proteins	0	0	4	0	0	0
atSmE-b	AT2G18740	Sm core Proteins	1	0	4	0	0	1
atSmF	AT4G30220	Sm core Proteins	0	0	1	0	0	0
atSmG-a	AT2G23930	Sm core Proteins	1	1	4	1	0	3
atSmG-b	AT3G11500	Sm core Proteins	0	0	4	0	2	3
atLSM2	AT1G03330	Sm core Proteins	0	1	1	0	1	1
atLSM3a	AT1G21190	Sm core Proteins	0	0	0	0	0	0
atLSM3b	AT1G76860	Sm core Proteins	0	0	1	0	0	0
atLSM4	AT5G27720	Sm core Proteins	0	0	0	1	2	2
atLSM5 /SAD1	AT5G48870	Sm core Proteins	0	0	0	0	0	0
atLSM6a	AT3G59810	Sm core Proteins	1	0	0	2	0	0
atLSM6b	AT2G43810	Sm core Proteins	1	2	2	0	1	0
atLSM7	AT2G03870	Sm core Proteins	1	0	1	0	0	0
atLSM8	AT1G65700	Sm core Proteins	0	0	0	0	0	0
atLSM1a	AT1G19120	Sm core Proteins	0	0	0	0	0	0
atLSM1b	AT3G14080	Sm core Proteins	0	0	0	0	0	0
atU1A	AT2G47580	U1 snRNP specific Proteins	0	0	0	0	0	0
atU1C	AT4G03120	U1 snRNP specific Proteins	0	0	0	0	0	0
atU1-70K	AT3G50670	U1 snRNP specific Proteins	0	1	0	0	0	2
atPrp39a	AT1G04080	U1 snRNP specific Proteins	1	1	1	1	2	0
atPrp39b	AT5G46400	U1 snRNP specific Proteins	0	0	0	0	0	0
atPrp40a	AT1G44910	U1 snRNP specific Proteins	2	0	3	0	0	3
atPrp40b	AT3G19670	U1 snRNP specific Proteins	0	0	0	0	0	1
atLuc7a	AT3G03340	U1 snRNP specific Proteins	0	0	0	0	0	4
atLuc7b	AT5G17440	U1 snRNP specific Proteins	3	0	1	2	2	4
atLuc7-rl	AT5G51410	U1 snRNP specific Proteins	2	0	2	0	0	1
atU2A	AT1G09760	U2 snRNP specific Proteins	4	1	11	2	2	6
atU2B"a	AT1G06960	U2 snRNP specific Proteins	1	0	0	0	2	2
atU2B"b	AT2G30260	U2 snRNP specific Proteins	0	2	3	2	0	0
atSAP114-1a	AT1G14650	U2 snRNP specific Proteins	4	1	11	0	2	3
atSAP114-1b	AT1G14640	U2 snRNP specific Proteins	0	0	0	0	0	0
atSAP114-2	AT5G06520	U2 snRNP specific Proteins	0	0	0	0	0	0
atSAP114-3	AT4G16200	U2 snRNP specific Proteins	0	0	0	0	0	0
atSAP114p	AT4G15580	U2 snRNP specific Proteins	0	0	0	0	0	0
atSAP61	AT5G06160	U2 snRNP specific Proteins	1	0	7	0	0	2
atSAP62	AT2G32600	U2 snRNP specific Proteins	0	1	6	0	0	1
atSAP130a	AT3G55200	U2 snRNP specific Proteins	0	0	0	0	0	0
atSAP130b	AT3G55220	U2 snRNP specific Proteins	3	2	12	1	3	4
atSF3b150	AT4G21660	U2 snRNP specific Proteins	0	0	7	0	0	0
atSF3b150p	AT1G11520	U2 snRNP specific Proteins	0	0	0	0	0	0
atSAP155	AT5G64270	U2 snRNP specific Proteins	5	11	24	2	7	7
atSAP49a	AT2G18510	U2 snRNP specific Proteins	0	1	4	1	2	3
atSAP49b	AT2G14550	U2 snRNP specific Proteins	0	0	0	0	0	0
atP14-1	AT5G12190	U2 snRNP specific Proteins	0	0	1	0	0	0
atP14-2	AT2G14870	U2 snRNP specific Proteins	0	0	0	0	0	0
SF3b 14b /PHP5A-a	AT1G07170	U2 snRNP specific Proteins	2	2	4	1	2	2
SF3b 14b /PHP5A-b	AT2G30000	U2 snRNP specific Proteins	0	0	0	0	0	0

SF3b10a	AT4G14342	U2 snRNP specific Proteins	0	0	0	0	0	0
SF3b10b	AT3G23325	U2 snRNP specific Proteins	1	1	2	0	0	1
atU5-15	AT5G08290	U5 snRNP specific Proteins	0	0	0	0	0	0
atU5-40	AT2G43770	U5 snRNP specific Proteins	2	0	17	3	1	5
atU5-100KD	AT2G33730	U5 snRNP specific Proteins	0	0	2	0	0	0
atU5-102KD	AT4G03430	U5 snRNP specific Proteins	5	5	14	3	0	8
atU5-116-1a	AT1G06220	U5 snRNP specific Proteins	12	9	50	0	7	24
atU5-116-1b	AT5G25230	U5 snRNP specific Proteins	0	0	0	0	0	0
atU5-116-2	AT1G56070	U5 snRNP specific Proteins	25	30	34	22	29	31
atU5-116-3	AT3G22980	U5 snRNP specific Proteins	0	1	0	1	2	1
atU5-200-1	AT5G61140	U5 snRNP specific Proteins	2	7	47	2	1	4
atU5-200-2a	AT1G20960	U5 snRNP specific Proteins	24	10	129	2	6	32
atU5-200-2b	AT2G42270	U5 snRNP specific Proteins	0	0	19	0	0	0
atU5-200-3	AT3G27730	U5 snRNP specific Proteins	0	0	0	0	0	0
atU5-220/Prp8a	AT1G80070	U5 snRNP specific Proteins	30	12	113	9	8	37
atU5-220/Prp8b	AT4G38780	U5 snRNP specific Proteins	0	0	0	0	0	0
atSAP90-1	AT1G28060	U4/U6 snRNP specific Proteins	0	0	0	1	1	2
atSAP90-2	AT3G55930	U4/U6 snRNP specific Proteins	0	0	0	0	0	0
atSAP90-3	AT3G56790	U4/U6 snRNP specific Proteins	0	0	0	0	0	0
atSAP60	AT2G41500	U4/U6 snRNP specific Proteins	0	0	4	2	0	1
atTri-20	AT2G38730	U4/U6 snRNP specific Proteins	2	2	2	1	1	2
atU5-61/Prp31a	AT1G60170	U4/U6 snRNP specific Proteins	0	1	8	0	0	1
atU5-61/Prp31b	AT3G60610	U4/U6 snRNP specific Proteins	0	0	0	0	0	0
atTri15.5-1a	AT5G20160	U4/U6 snRNP specific Proteins	1	1	1	0	0	2
atTri15.5-1b	AT4G12600	U4/U6 snRNP specific Proteins	0	0	0	0	0	0
atTri15.5-1c	AT4G22380	U4/U6 snRNP specific Proteins	0	0	0	0	0	0
atTri65a	AT4G22350	Tri-snRNP Specific (U4/U6.U5) Proteins	0	0	0	0	0	0
atTri65b	AT4G22290	Tri-snRNP Specific (U4/U6.U5) Proteins	0	0	0	0	0	0
atTri65c	AT4G22410	Tri-snRNP Specific (U4/U6.U5) Proteins	0	0	0	0	0	0
atTri110	AT5G16780	Tri-snRNP Specific (U4/U6.U5) Proteins	1	0	1	0	1	1
atTri-27kD/RY1	AT5G57370	Tri-snRNP Specific (U4/U6.U5) Proteins	0	0	0	0	0	0
atSnu23	AT3G05760	Tri-snRNP Specific (U4/U6.U5) Proteins	0	0	0	0	0	0
atU11/U12-35kD	AT2G43370	18S U11/U12 snRNP Specific Proteins	0	0	0	0	0	0
atU11/U12-25K	AT3G07860	18S U11/U12 snRNP Specific Proteins	0	0	0	0	0	0
atU11/U12-65K	AT1G09230	18S U11/U12 snRNP Specific Proteins	0	0	0	0	0	1
atU11/U12-31K	AT3G10400	18S U11/U12 snRNP Specific Proteins	0	0	0	0	0	0
atU2AF35a/AUSa	AT1G27650	Splice site selection Proteins	2	1	0	0	1	2
atU2AF35/AUSb	AT5G42820	Splice site selection Proteins	0	0	2	0	0	0
atU2AF65b/AULa	AT1G60900	Splice site selection Proteins	1	0	0	0	1	1
atU2AF65a/AULb	AT4G36690	Splice site selection Proteins	0	1	1	0	0	0
AUL2	AT2G33440	Splice site selection Proteins	0	0	0	0	0	0
AUL3p	AT1G60830	Splice site selection Proteins	0	0	0	0	0	0
atUrp	AT1G10320	Splice site selection Proteins	0	0	0	0	0	0
atSF1/BBP	AT5G51300	Splice site selection Proteins	0	0	0	1	1	1
atCBP20	AT5G44200	Splice site selection Proteins	0	0	1	0	0	0
atCBP80	AT2G13540	Splice site selection Proteins	1	0	0	1	0	0
atPTB1	AT1G43190	Splice site selection Proteins	5	5	4	2	3	3
atPTB2a	AT3G01150	Splice site selection Proteins	0	0	1	0	0	0
atPTB2b	AT5G53180	Splice site selection Proteins	0	1	4	1	0	1
atSC35	AT5G64200	SR Proteins	0	0	0	1	1	1
atSR33/atSCL33	AT1G55310	SR Proteins	0	0	0	0	0	1
atSCL30a	AT3G13570	SR Proteins	0	0	0	0	0	1
atSCL30	AT3G55460	SR Proteins	0	0	0	0	0	1
atSCL28	AT5G18810	SR Proteins	0	0	0	0	0	0
atSR1/atSRp34	AT1G02840	SR Proteins	6	1	1	3	2	7
atSRp34a	AT3G49430	SR Proteins	6	0	0	4	4	8
atSRp34b	AT4G02430	SR Proteins	0	0	0	0	0	0
atSRp30	AT1G09140	SR Proteins	0	0	0	0	0	0

atRSZp22/atSRZ22	AT4G31580	SR Proteins	4	3	3	2	4	3
atRSZp22a	AT2G24590	SR Proteins	4	0	3	2	5	3
atRSZp21/atSRZ21	AT1G23860	SR Proteins	2	0	3	0	0	0
atRSZ33	AT2G37340	SR Proteins	1	0	0	0	0	1
atRSZ34	AT3G53500	SR Proteins	1	0	0	0	0	0
atRSp31a	AT2G46610	SR Proteins	0	0	0	0	0	0
atRSp31	AT3G61860	SR Proteins	0	0	0	0	0	0
atRSp41	AT5G52040	SR Proteins	1	0	1	13	0	4
atRSp40/atRSP35	AT4G25500	SR Proteins	0	0	0	6	1	3
atPrp43-1	AT5G14900	17S U2 associated Proteins	0	0	0	0	0	0
atPrp43-2a	AT3G62310	17S U2 associated Proteins	5	0	0	2	2	3
atPrp43-2b	AT2G47250	17S U2 associated Proteins	0	2	1	2	0	0
atSR140-1	AT5G25060	17S U2 associated Proteins	0	0	0	0	4	4
atSR140-2	AT5G10800	17S U2 associated Proteins	0	0	0	0	0	0
atSPF45	AT1G30480	17S U2 associated Proteins	0	0	0	0	0	0
atSPF30	AT2G02570	17S U2 associated Proteins	1	0	0	0	0	0
atPrp19a	AT1G04510	35S U5 associated Proteins	5	4	13	1	6	8
atPrp19b	AT2G33340	35S U5 associated Proteins	8	5	15	2	4	9
atCDC5	AT1G09770	35S U5 associated Proteins	12	1	41	0	0	21
atPRL1	AT4G15900	35S U5 associated Proteins	2	1	9	0	0	5
atPRL2	AT3G16650	35S U5 associated Proteins	0	0	3	0	0	0
atAD-002	AT3G13200	35S U5 associated Proteins	3	1	11	0	1	6
HSP73-1	AT3G12580	35S U5 associated Proteins	14	24	22	11	15	0
HSP73-2	AT5G42020	35S U5 associated Proteins	24	35	26	16	29	27
HSP73-3	AT5G02500	35S U5 associated Proteins	24	33	33	18	28	23
atSPF27	AT3G18165	35S U5 associated Proteins	2	0	8	1	1	2
atCTNNNBL1	AT3G02710	35S U5 associated Proteins	0	0	0	1	1	1
atSyf1	AT5G28740	35S U5 associated Proteins	14	2	33	2	7	23
atCRN1a	AT5G45990	35S U5 associated Proteins	0	0	0	0	0	0
atCRN1b	AT3G13210	35S U5 associated Proteins	0	0	0	0	0	0
atCRN1c	AT5G41770	35S U5 associated Proteins	7	2	24	0	2	15
atCRN2	AT3G51110	35S U5 associated Proteins	0	0	0	0	0	0
atIsy1	AT3G18790	35S U5 associated Proteins	2	1	7	2	2	6
atGCIPp29	AT2G16860	35S U5 associated Proteins	0	0	3	1	0	6
atSKIP	AT1G77180	35S U5 associated Proteins	4	0	16	1	1	12
atECM2-1a	AT1G07360	35S U5 associated Proteins	4	1	11	0	0	4
atECM2-1b	AT2G29580	35S U5 associated Proteins	0	0	0	3	0	0
atECM2-2	AT5G07060	35S U5 associated Proteins	0	0	3	0	0	0
atAquarius	AT2G38770	35S U5 associated Proteins	9	4	31	3	1	21
atMGC23918	AT3G05070	35S U5 associated Proteins	0	0	6	0	0	5
atG10	AT4G21110	35S U5 associated Proteins	1	1	2	0	0	1
atCypE1a/CYP2	AT2G21130	35S U5 associated Proteins	0	2	1	2	2	2
atCypE1b	AT4G38740	35S U5 associated Proteins	3	0	0	0	0	0
atCypE2a/ROC3	AT2G16600	35S U5 associated Proteins	0	0	5	0	0	0
atCypE2b	AT4G34870	35S U5 associated Proteins	4	4	4	5	4	4
atPPIase-like1	AT2G36130	35S U5 associated Proteins	1	0	7	0	0	2
atNPW38	AT2G41020	BDU1 specific Proteins	0	0	0	0	0	0
atN-CoR1	AT3G52250	BDU1 specific Proteins	0	0	0	0	0	0
atPRP4K-1	AT3G25840	BDU1 specific Proteins	0	1	0	0	0	0
atPRP4K-2	AT1G13350	BDU1 specific Proteins	0	0	1	0	0	0
atPRP4K-3	AT3G53640	BDU1 specific Proteins	0	0	0	0	0	0
atFBP21	AT1G49590	BDU1 specific Proteins	0	0	2	0	0	0
atTBL1-rp1	AT5G67320	BDU1 specific Proteins	0	0	0	0	0	0
atSmc1	AT3G54670	BDU1 specific Proteins	1	0	0	1	2	6
atALY-1a	AT5G02530	Exon junction complex (EJC) Proteins	3	0	0	0	1	1
atALY-1b	AT5G59950	Exon junction complex (EJC) Proteins	3	1	1	0	1	1
atALY-2a	AT5G37720	Exon junction complex (EJC) Proteins	2	0	0	0	0	3
atALY-2b	AT1G66260	Exon junction complex (EJC) Proteins	0	0	0	0	0	0

atY14	AT1G51510	Exon junction complex (EJC) Proteins	2	1	0	1	1	1
atSRM102	AT2G29210	Exon junction complex (EJC) Proteins	0	0	0	0	1	3
atMagoh	AT1G02140	Exon junction complex (EJC) Proteins	2	3	2	2	1	1
atDDX48/eIF4A3-1	AT3G19760	Exon junction complex (EJC) Proteins	9	9	21	6	7	8
atDDX48/eIF4A3-2	AT1G51380	Exon junction complex (EJC) Proteins	0	0	0	0	0	0
atSR45/atRNPS1	AT1G16610	Exon junction complex (EJC) Proteins	1	0	0	0	1	3
atUAP56a	AT5G11200	Exon junction complex (EJC) Proteins	6	5	13	4	3	5
atUAP56b	AT5G11170	Exon junction complex (EJC) Proteins	0	0	0	0	0	0
atPinin	AT1G15200	Exon junction complex (EJC) Proteins	1	0	0	0	0	3
atPrp22-1	AT3G26560	Second step splicing Proteins	2	0	1	1	1	5
atPrp22-2	AT1G26370	Second step splicing Proteins	0	0	0	0	0	0
atPrp22-3	AT1G27900	Second step splicing Proteins	0	0	0	1	0	1
atPrp17-1	AT1G10580	Second step splicing Proteins	2	1	12	0	0	5
atPrp17-2	AT5G54520	Second step splicing Proteins	0	0	0	0	0	0
atPrp18-1	AT1G03140	Second step splicing Proteins	2	2	1	2	2	4
atPrp18-2	AT1G54590	Second step splicing Proteins	0	0	0	0	0	0
atSLU7-1a	AT1G65660	Second step splicing Proteins	0	0	5	0	0	0
atSLU7-1b	AT4G37120	Second step splicing Proteins	0	0	8	0	34	0
atSLU7-2	AT3G45950	Second step splicing Proteins	0	0	0	0	1	0
atPrp16	AT5G13010	Second step splicing Proteins	0	0	2	1	2	2
atSRM300like	AT3G23900	Other known splicing Proteins	2	0	0	0	1	6
atTra/SFRS1	AT1G07350	Other known splicing Proteins	0	0	0	0	0	0
atPrp2-1a	AT1G32490	Other known splicing Proteins	1	0	4	0	0	1
atPrp2-1b	AT2G35340	Other known splicing Proteins	0	0	0	0	0	0
atPrp2-2	AT4G16680	Other known splicing Proteins	0	0	0	0	0	0
atPrp5-1a	AT3G09620	Other known splicing Proteins	0	0	0	0	0	0
atPrp5-1b	AT1G20920	Other known splicing Proteins	0	0	0	2	1	3
atPrp5-2	AT2G47330	Other known splicing Proteins	0	0	0	0	0	0
atDbr1	AT4G31770	Other known splicing Proteins	0	0	0	1	0	0
AFC1	AT3G53570	SR kinase Proteins	0	0	0	0	0	0
AFC2	AT4G24740	SR kinase Proteins	0	0	0	0	0	0
AFC3	AT4G32660	SR kinase Proteins	0	0	0	0	0	0
atSRPK1a	AT2G17530	SR kinase Proteins	0	0	0	0	0	0
atSRPK1b	AT4G35500	SR kinase Proteins	0	0	0	0	0	1
atSRPK2a	AT5G22840	SR kinase Proteins	0	0	0	0	0	2
atSRPK2b	AT3G53030	SR kinase Proteins	0	0	0	1	0	2
atSRPK2c	AT3G44850	SR kinase Proteins	0	0	0	0	0	0
atGRBP1a	AT1G18630	Glycine-Rich RNA binding Proteins	0	2	2	1	1	1
atGRBP1b	AT1G74230	Glycine-Rich RNA binding Proteins	1	1	1	1	1	1
atGRBP1c	AT4G13850	Glycine-Rich RNA binding Proteins	2	2	1	3	3	3
atGRBP1d	AT3G23830	Glycine-Rich RNA binding Proteins	0	0	2	1	0	0
atGRBP1e	AT5G61030	Glycine-Rich RNA binding Proteins	0	0	0	1	0	0
atGRBP2	AT2G16260	Glycine-Rich RNA binding Proteins	0	0	0	0	0	0
AtGRP7/atGRBP3a	AT2G21660	Glycine-Rich RNA binding Proteins	9	7	8	8	6	6
AtGRP8/atGRBP3b	AT4G39260	Glycine-Rich RNA binding Proteins	6	4	5	4	6	5
AtRNPA/B_1	AT4G14300	hnRNP A/B family Proteins	0	0	2	0	0	0
AtRNPA/B_2	AT2G33410	hnRNP A/B family Proteins	0	0	1	1	0	0
AtRNPA/B_3	AT5G55550	hnRNP A/B family Proteins	0	0	0	0	0	0
AtRNPA/B_4	AT4G26650	hnRNP A/B family Proteins	0	0	5	2	0	0
AtRNPA/B_5	AT5G47620	hnRNP A/B family Proteins	0	0	0	0	0	0
AtRNPA/B_6	AT3G07810	hnRNP A/B family Proteins	0	0	1	3	2	1
AtRNPA/B_7	AT1G58470	hnRNP A/B family Proteins	0	0	0	0	0	0
AtRNPA/B_8a	AT5G40490	hnRNP A/B family Proteins	1	0	3	1	1	1
AtRNPA/B_8b	AT1G17640	hnRNP A/B family Proteins	0	0	0	0	0	0
AtRNP_N1	AT3G13224	hnRNP A/B family Proteins	0	0	0	1	1	0
UBA2a	AT3G56860	hnRNP A/B family Proteins	2	2	3	3	1	1
UBA2b	AT2G41060	hnRNP A/B family Proteins	0	0	0	0	0	0
UBA2c	AT3G15010	hnRNP A/B family Proteins	2	0	0	1	0	1

at-hnRNP-E	AT3G04610	Other hnRNP (with animal homologs) Proteins	3	2	3	1	2	2
at-hnRNP-F/AtRNPH/F_	AT5G66010	Other hnRNP (with animal homologs) Proteins	0	0	0	0	0	0
at-hnRNP-H/AtRNPH/F_	AT3G20890	Other hnRNP (with animal homologs) Proteins	0	0	0	0	0	0
at-hnRNP-G1	AT5G04280	Other hnRNP (with animal homologs) Proteins	4	2	0	1	3	2
at-hnRNP-G2	AT3G26420	Other hnRNP (with animal homologs) Proteins	0	0	3	0	0	0
at-hnRNP-G3	AT1G60650	Other hnRNP (with animal homologs) Proteins	0	0	0	0	0	1
at-hnRNP-P	AT1G50300	Other hnRNP (with animal homologs) Proteins	0	0	0	0	0	1
hnRNP-R1	AT4G00830	Other hnRNP (with animal homologs) Proteins	0	0	0	0	0	2
hnRNP-R2	AT3G52660	Other hnRNP (with animal homologs) Proteins	0	0	0	0	1	1
hnRNP-R3 / AtRNPA/B_	AT2G44710	Other hnRNP (with animal homologs) Proteins	0	0	0	0	0	0
AtCUG-BP1	AT4G03110	Other hnRNP (with animal homologs) Proteins	1	3	4	0	2	1
AtCUG-BP2	AT1G03457	Other hnRNP (with animal homologs) Proteins	0	0	0	0	0	0
atFCA1	AT4G16280	Other hnRNP (with animal homologs) Proteins	0	0	0	0	0	0
atFCA2	AT2G47310	Other hnRNP (with animal homologs) Proteins	0	0	0	0	0	0
AtUBP1a	AT1G54080	Other plant hnRNPs Proteins	0	3	6	0	3	1
AtUBP1c	AT3G14100	Other plant hnRNPs Proteins	3	0	8	2	0	0
AtUBP1b	AT1G17370	Other plant hnRNPs Proteins	0	3	6	3	3	0
UBA1a	AT2G22090	Other plant hnRNPs Proteins	0	0	0	0	0	0
UBA1b	AT2G22100	Other plant hnRNPs Proteins	0	0	0	0	0	1
UBA1c	AT2G19380	Other plant hnRNPs Proteins	0	0	0	0	0	0
atRBP45a	AT5G54900	Other plant hnRNPs Proteins	2	3	3	2	2	3
atRBP45c	AT4G27000	Other plant hnRNPs Proteins	3	4	3	2	3	4
AtRBP45b	AT1G11650	Other plant hnRNPs Proteins	2	1	5	2	2	1
atRBP45d	AT5G19350	Other plant hnRNPs Proteins	0	0	0	2	1	1
AtRBP47a	AT1G49600	Other plant hnRNPs Proteins	0	4	6	2	3	0
AtRBP47b	AT3G19130	Other plant hnRNPs Proteins	2	0	0	3	4	3
AtRBP47c	AT1G47490	Other plant hnRNPs Proteins	0	0	0	0	0	0
AtRBP47c	AT1G47500	Other plant hnRNPs Proteins	1	3	4	2	5	4
Ath1	AT4G16830	Other plant hnRNPs Proteins	2	1	0	1	1	1
Ath2	AT4G17520	Other plant hnRNPs Proteins	6	0	0	1	3	4
Ath3	AT5G47210	Other plant hnRNPs Proteins	7	3	2	5	9	7
atCPSF-160k	AT5G51660	other processes Proteins	0	1	1	2	2	2
atCFI-25kD-1	AT4G25550	other processes Proteins	1	1	3	0	0	2
atCFI-25kD-2	AT4G29820	other processes Proteins	0	0	0	0	0	0
at-eIF3sub6	AT3G57290	other processes Proteins	11	14	16	5	10	11
at-eIF3sub2a	AT2G46280	other processes Proteins	7	9	12	6	5	7
at-eIF3sub2b	AT2G46290	other processes Proteins	0	0	0	0	0	0
atTHO2	AT1G24706	other processes Proteins	0	0	1	1	1	2
atHPR1	AT5G09860	other processes Proteins	1	0	0	0	0	0
atKIAA0983-1	AT5G42920	other processes Proteins	0	0	2	1	1	1
atKIAA0983-2	AT1G45233	other processes Proteins	0	0	0	0	0	0
atC21orf66	AT5G08550	other processes Proteins	1	1	1	0	0	1
atKin17-1	AT1G55460	other processes Proteins	0	0	0	0	0	0
atKin17-2	AT5G51795	other processes Proteins	0	0	0	0	0	0
atPABP1/atPAB6	AT3G16380	Poly A binding Proteins	0	0	0	0	0	0
atPABP2/atPAB2	AT4G34110	Poly A binding Proteins	4	5	8	7	10	9
atPABP3/atPAB3	AT1G22760	Poly A binding Proteins	0	0	0	0	0	0
atPABP4/atPAB4	AT2G23350	Poly A binding Proteins	7	5	11	5	8	7
atPABP5/atPAB5	AT1G71770	Poly A binding Proteins	0	0	0	0	0	0
atPABP6/atPAB7	AT2G36660	Poly A binding Proteins	0	0	0	0	0	0
atPABP7/atPAB8	AT1G49760	Poly A binding Proteins	8	5	9	6	9	10
atPABP-like/atPAB1	AT1G34140	Poly A binding Proteins	0	0	0	0	0	0
atDbp5	AT3G53110	DEAD/H box helixase Proteins	4	4	10	4	4	5
atAbstrakt-a	AT5G51280	DEAD/H box helixase Proteins	11	64	0	0	11	10
atAbstrakt-b	AT4G33370	DEAD/H box helixase Proteins	0	15	0	0	0	0
atDDX35	AT4G18465	DEAD/H box helixase Proteins	0	0	3	2	0	2
atKIAA0052a	AT1G59760	DEAD/H box helixase Proteins	0	0	0	0	0	0
atKIAA0052b	AT2G06990	DEAD/H box helixase Proteins	0	0	1	1	1	0

p72-1	AT1G55150	DEAD/H box helixase Proteins	3	0	0	2	0	0
p72-2	AT5G63120	DEAD/H box helixase Proteins	2	0	0	0	0	0
p72-3	AT3G06480	DEAD/H box helixase Proteins	0	0	0	0	0	2
p72-4	AT3G01540	DEAD/H box helixase Proteins	5	3	10	3	3	4
p72-5	AT5G14610	DEAD/H box helixase Proteins	0	2	0	0	0	0
atKIA0073	AT3G44600	cis-trans prolyl isomerases Proteins	0	0	0	1	1	0
atCYP-60	AT5G67530	cis-trans prolyl isomerases Proteins	0	0	0	0	0	0
atPPIL3	AT1G01940	cis-trans prolyl isomerases Proteins	1	1	1	0	3	1
atSDCCAG10	AT4G33060	cis-trans prolyl isomerases Proteins	0	0	0	0	0	0
atCA150	AT3G19840	Related to spliceosome Proteins	0	0	0	0	0	0
atKIAA1604	AT1G80930	Related to spliceosome Proteins	6	1	11	0	0	12
atMGC13125	AT1G31870	Related to spliceosome Proteins	0	0	0	0	0	0
atMFAP1a	AT5G17900	Related to spliceosome Proteins	0	0	0	0	0	0
atMFAP1b	AT4G08580	Related to spliceosome Proteins	0	0	0	0	0	0
atRED	AT2G26460	Related to spliceosome Proteins	0	0	0	0	0	0
atNOSIP	AT1G61620	Related to spliceosome Proteins	3	0	1	1	0	2
atDGCR14	AT3G07790	Related to spliceosome Proteins	0	0	4	0	0	2
atCactin	AT1G03910	Related to spliceosome Proteins	3	13	0	1	1	35
atTFIP11/TIP39a	AT1G17070	Related to spliceosome Proteins	0	0	1	0	1	1
atTFIP11/TIP39b	AT2G42330	Related to spliceosome Proteins	0	0	0	0	0	0
atSmu-1	AT1G73720	Related to spliceosome Proteins	0	0	0	1	1	1
atFLJ10206	AT5G23080	Related to spliceosome Proteins	0	0	0	0	0	1
atPSP1	AT1G67210	Related to spliceosome Proteins	0	0	0	0	0	0
atPSP2	AT5G38600	Related to spliceosome Proteins	0	1	0	0	1	1
atCWF16-1	AT1G17130	Related to spliceosome Proteins	0	0	0	0	0	0
atCWF16-2	AT3G43250	Related to spliceosome Proteins	0	0	0	0	0	0
atCWF16-3	AT2G32050	Related to spliceosome Proteins	0	0	0	0	0	0
atZNF183a	AT1G01350	Related to spliceosome Proteins	1	1	1	0	0	0
atZNF183b	AT5G06420	Related to spliceosome Proteins	0	0	0	0	0	0
atCAJ1/FLJ10634	AT5G23590	Related to spliceosome Proteins	0	0	0	0	0	0
atSPF31/Jiv	AT5G22080	Related to spliceosome Proteins	1	1	0	0	2	3
atSF4	AT3G52120	Related to spliceosome Proteins	1	0	0	0	1	1
atRBM5	AT3G54230	Related to spliceosome Proteins	0	0	0	0	0	0
atCP33/PUF60like	AT3G52380	Related to spliceosome Proteins	2	2	6	4	4	3
atPMSCL2-1	AT5G35910	Related to spliceosome Proteins	0	0	0	0	0	0
atPMSCL2-2	AT1G54440	Related to spliceosome Proteins	0	0	0	0	0	0
atPMSCL2-3	AT2G32415	Related to spliceosome Proteins	0	0	0	1	0	1
atNPC2a	AT5G09880	Related to spliceosome Proteins	0	0	0	0	1	1
atNPC2b	AT2G16940	Related to spliceosome Proteins	2	2	2	2	2	2
atIMP3/KOC	AT5G15270	Related to spliceosome Proteins	1	1	2	1	1	3
atAcinusL	AT4G39680	Related to spliceosome Proteins	1	0	0	0	0	0
atCIP1/atNuMA	AT5G41790	Related to spliceosome Proteins	34	5	9	2	6	24
atUBL5a	AT5G42300	Related to spliceosome Proteins	0	0	0	0	0	0
atUBL5b	AT3G45180	Related to spliceosome Proteins	1	0	0	0	0	0
atRuvB/TIP48a	AT3G49830	Related to spliceosome Proteins	0	0	0	0	0	0
atRuvB/TIP48b	AT5G67630	Related to spliceosome Proteins	4	4	12	4	3	5
atTIP49	AT5G22330	Related to spliceosome Proteins	5	4	8	2	3	6
atCDCL2p110-1	AT1G67580	Related to spliceosome Proteins	0	0	0	1	0	2
atCDCL2p110-2	AT5G63370	Related to spliceosome Proteins	0	0	0	0	0	0
atKIAA0853	AT5G53440	Related to spliceosome Proteins	0	1	0	1	1	1
atTPX2	AT1G03780	Related to spliceosome Proteins	0	0	0	0	0	0
atRACK1a	AT3G18130	Related to spliceosome Proteins	3	2	5	3	2	0
atRACK1b	AT1G48630	Related to spliceosome Proteins	2	2	0	3	2	2
atRACK1c/ARCA	AT1G18080	Related to spliceosome Proteins	2	2	5	4	3	4
atDKFZP586O0120	AT1G16810	Related to spliceosome Proteins	0	0	0	0	0	0
atMORG1	AT5G64730	Related to spliceosome Proteins	0	0	0	0	0	1
atKu70	AT1G16970	Related to spliceosome Proteins	0	0	0	0	0	0
atSFL1	AT4G35785	Splicing factor like Proteins	0	0	0	0	0	0

atSFL2	AT5G59860	Splicing factor like Proteins	0	0	0	0	0	0
atSFL3	AT2G25970	Splicing factor like Proteins	5	2	1	2	4	4
atSFL4	AT2G40650	Splicing factor like Proteins	0	0	2	0	1	1
atSFL5	AT3G11960	Splicing factor like Proteins	1	1	0	1	0	0
atSFL6a	AT3G63400	Splicing factor like Proteins	1	0	0	0	0	2
atSFL6b	AT3G56070	Splicing factor like Proteins	0	0	0	2	0	0
atRBD1a	AT3G08620	Nucleotide Binding Proteins	0	0	0	0	0	0
atRBD1b	AT2G38610	Nucleotide Binding Proteins	0	2	4	0	0	0
atRBD2a	AT1G20880	Nucleotide Binding Proteins	0	0	0	0	0	0
atRBD2b	AT5G58470	Nucleotide Binding Proteins	2	0	0	0	0	0
atRBD3	AT5G28330	Nucleotide Binding Proteins	0	0	0	0	0	0
atRBD4	AT5G28390	Nucleotide Binding Proteins	0	0	0	0	0	0
atRBD5	AT5G06210	Nucleotide Binding Proteins	0	0	0	0	0	0
atCP29	AT3G53460	Nucleotide Binding Proteins	5	3	5	5	5	5
atCP31a	AT4G24770	Nucleotide Binding Proteins	2	3	5	4	2	3
atCP31b	AT5G50250	Nucleotide Binding Proteins	0	0	0	3	3	3
atDBP1a	AT3G05060	Nucleotide Binding Proteins	7	7	9	2	8	9
atDBP1b	AT5G27120	Nucleotide Binding Proteins	6	6	6	1	3	6
atDBP1c	AT5G27140	Nucleotide Binding Proteins	0	0	0	0	0	0
atDBP2a	AT4G21100	Nucleotide Binding Proteins	0	0	0	2	0	4
atDBP2b	AT4G05420	Nucleotide Binding Proteins	1	3	1	3	1	4
atEF1	AT1G62750	Other multiple functional Proteins	11	12	18	13	11	8
atEF2	AT1G09660	Other multiple functional Proteins	0	0	1	0	0	0
atEFMa	AT2G45030	Other multiple functional Proteins	0	0	0	0	0	0
atEFMb	AT1G45332	Other multiple functional Proteins	0	1	0	2	1	0
atSPT5a	AT4G08350	Other multiple functional Proteins	1	2	2	5	2	3
atSPT5b	AT2G34210	Other multiple functional Proteins	0	0	0	0	0	0
AtGRP17	AT5G07530	Glycine rich Proteins	0	0	0	0	0	0
AtGRP16	AT5G07540	Glycine rich Proteins	0	0	0	0	0	0
AtGRP14	AT5G07510	Glycine rich Proteins	0	0	0	0	0	0
AtGRP18	AT5G07520	Glycine rich Proteins	0	0	0	0	0	0
AtGRP19	AT5G07550	Glycine rich Proteins	0	0	0	0	0	0
AtGRP20	AT5G07560	Glycine rich Proteins	0	0	0	0	0	0
atGRPs-1	AT4G30460	Glycine rich Proteins	0	0	0	0	0	0
atGRPs-2	AT4G36230	Glycine rich Proteins	0	0	0	0	0	0
atGRPs-3	AT3G17110	Glycine rich Proteins	0	0	0	0	0	0
atGRPs-4	AT5G49350	Glycine rich Proteins	0	0	0	0	0	0
AtGRP5	AT3G20470	Glycine rich Proteins	0	0	0	0	0	0
atGRPs-6a	AT1G04800	Glycine rich Proteins	0	0	0	0	0	0
atGRPs-6b	AT2G32690	Glycine rich Proteins	0	0	0	0	0	0
atGRPs-7	AT2G30560	Glycine rich Proteins	0	0	0	0	0	0
atGRPs-8a	AT3G17050	Glycine rich Proteins	0	0	0	0	0	0
atGRPs-8b	AT3G23450	Glycine rich Proteins	4	0	0	2	2	2
atGRPs-8c	AT4G22020	Glycine rich Proteins	0	0	0	0	0	0
atGRPs-9	AT5G46730	Glycine rich Proteins	0	0	0	0	0	0
atGRPs-10	AT2G36120	Glycine rich Proteins	0	0	0	0	0	0
atGRPs-11a	AT2G05580	Glycine rich Proteins	0	0	0	0	0	0
atGRPs-11b	AT5G35660	Glycine rich Proteins	0	0	0	0	0	0
atGRPs-12a	AT4G22500	Glycine rich Proteins	0	0	0	0	0	0
atGRPs-12b	AT4G22480	Glycine rich Proteins	0	0	0	0	0	0
DCS1	AT5G53800		36	0	0	0	1	4
DCS2	AT5G51280		11	64	0	0	11	10
DCS3	AT1G80070		30	12	113	9	8	37
DCS4	AT4G01020		0	0	0	54	0	0
DCS5	AT4G37120		0	0	8	0	34	0
DCS6	AT1G03910		3	13	0	1	1	35

Supplementary Table 3. Primers used in the research.

Primer's name	Primer sequences (5'→3')	
**Plasmid constructs**	**Underscored nucleotides indicate restriction sites for cloning**	**Construct's name**
ABA3-attB1-F	GGGGACAAGTTTGTACAAAAAAGCAGGCTTCATGGAAGCATTTCTTAAGGAAT	*pDONR104-ABA3*
ABA3-attB2-R	GGGGACCACTTTGTACAAGAAAGCTGGGTCTTATTCAATATCTGGATTAACTTCT	
ABA3(DT1-BsF)	ATATATGGTCTCGATTGACAAGCTTCAGCGCTGCTGGTT	*pHEE401E-ABA3*
ABA3(DT1-F0)	TGACAAGCTTCAGCGCTGCTGGTTTTAGAGCTAGAAATAGC	
ABA3(DT2-R0)	AACCGTTTCCATGTCGTAAAGCCAATCTCTTAGTCGACTCTAC	
ABA3(DT2-BsR)	ATTATTGGTCTCGAAACCGTTTCCATGTCGTAAAGCC	
DCS1-attB1-F	GGGGACAAGTTTGTACAAAAAAGCAGGCTTCATGGGAAGTGACCGCGACGGTG	*pDONR104-DCS1 / pDONR203-DCS1-2*
DCS1-attB2-R	GGGGACCACTTTGTACAAGAAAGCTGGGTCTCACATGGGAACATCCGGATCC	
DCS2-attB1-F	GGGGACAAGTTTGTACAAAAAAGCAGGCTTCATGGAATCCATCATGGAAGAAGC	*pDONR104-DCS2 / pDONR203-DCS2-1*
DCS2-attB2-R	GGGGACCACTTTGTACAAGAAAGCTGGGTCTTATATTTCTCCTCTGTAGCCACCC	*/pDONR104-DCS2-1*
DCS3(SalI)-F	TGATTTCAGAAGAGGATCTAGTCGACATGTGGAACAACAACGATGGTATG	*pCambia1300-35Spro:myc-gDCS3-1*
DCS3(SalI)-R	AGAAGCTTGCATGCCTGCAGGTCGACTCAAGTAAATGTGTCCTCGCGATC	
DCS4(BamHI)-F	GCGGCCGCTCTAGAACTAGTGGATCCATGAGGAACTCTTTTCCTCCTTC	*pCambia1307-35Spro:DCS4-1-flag*
DCS4(HindIII)-R	TTGTAGTCGACGGTATCGATAAGCTTAAGCCTGTGAACAGGCTGGG	
DCS4(NcoI)-F	TTTTCTGATTAACAGCCATGGCTGACTACAAAGACGATGACGACAAAATGAGGAACTCTTTTCCTCCTTC	*pCambia1300-UBQ10pro:flag-DCS4*
DCS4(PstI)-R	ATACGAACGAAAGCTCTGCAGTCAAAGCCTGTGAACAGGCTGGG	
DCS5-attB1-F	GGGGACAAGTTTGTACAAAAAAGCAGGCTGGATGGCGACGGCTTCAG	*pDONR104-DCS5 / pDONR104-DCS5-1*
DCS5-attB2-R	GGGGACCACTTTGTACAAGAAAGCTGGGTGGCCGGGGAAATTTCTCATTG	
DCS6-attB1-F	GGGGACAAGTTTGTACAAAAAAGCAGGCTTCATGGGTTCTCATGGTAAGGGTAAG	*pDONR104-gDCS6 / pDONR104-DCS6-1*
DCS6-attB2-R	GGGGACCACTTTGTACAAGAAAGCTGGGTCTTATCGCCTGTACCTATGTCGTTTA	*/pDONR203-DCS6-1*
His-DCS1(EcoRI)-F	ATGGGTCGCGGATCCGAATTCATGGGAAGTGACCGCGACGGTG	*pET28a-DCS1(1-97AA) / pET28a-DCS1*
His-DCS1(97AA)(XhoI)-R	GTGGTGGTGGTGGTGCTCGAGTTCACCTACTCCTAGATGATTTCCG	
His-DCS1(XhoI)-R	GTGGTGGTGGTGGTGCTCGAGTCATGGGAACATCCGGATCCAAC	
DCS6-RNAi-1F	GCGGCCGCTCTAGAACTAGTGGATCCATGGGTTCTCATGGTAAGGGTAAG	*pCambia1307-DCS6-RNAi*
DCS6-RNAi-1R	TTCCATGAGGTTGCTTCTGACGCGAAGCTCTTCCATATCT	
intron-F	TCAGAAGCAACCTCATGGAA	
intron-R	GCTGTTATGTTCAGTGTCAAGCTG	
DCS6-RNAi-2F	CAGCTTGACACTGAACATAACAGCCGCGAAGCTCTTCCATATCT	
DCS6-RNAi-2F	TTGTAGTCGACGGTATCGATAAGCTTATGGGTTCTCATGGTAAGGG	
**Real-time qPCR**		
PIF4(AS-qRT)-F	GGTAAGGCCAATACGAAGCC	
PIF4(AS-qRT)-R	ACGGTTGTTGACTTTGCTGT	
PIF4(total-qRT)-F	CCTCCAGAGACCAACCTCAG	
PIF4(total-qRT)-R	GACTTGAGGAGGAGGGTCAG	
RVE1(AS-qRT)-F	AACTTCTGAGCATGGCTTCG	
RVE1(AS-qRT)-R	GCCTTACCTGGACCCTCAAA	
RVE1(total-qRT)-F	CAAGCAGATACAATTCAATGACC	
RVE1(total-qRT)-R	CGTATAATTTCAAGGCTTCAAC	
ABA3(AS-qRT)-F	GCCACCAGTGACCTTATAGCG	
ABA3(AS-qRT)-R	AAGATTGATTGAAACATATGGAAC	
ABA3(total-qRT)-F	CCGTGGACCCAAGACAGTAA	
ABA3(total-qRT)-R	TAAGCTGGCCTGGTTGGTTA	
HRD1B(AS-qRT)-F	TGCATCATACAGCCTAGCTT	
HRD1B(AS-qRT)-R	AGTCACGAACAAGCTCCAGA	
HRD1B(total-qRT)-F	ACCCAGCGACTGTGTATCTA	
HRD1B(total-qRT)-R	AGCAAAGAGAATCTCCATCAGT	
PP2A(qRT)-F	TATCGGATGACGATTCTTCGTGCAG	
PP2A(qRT)-R	GCTTGGTCGACTATCGGAATGAGAG	
**Semi-quantitative PCR**		
PIF4(AS-RT)-F	GAGTTCAACCTCAGCAGTTCATAC	
PIF4(AS-RT)-R	GTAAAAAAGTGGCTCACCAACCTA	

RVE1(AS-RT)-F	GTACGGAAGCCATACACGATAACA
RVE1(AS-RT)-R	CTGAGCATGGCTTCGAATCTGA
ABA3(AS-RT)-F	ACTGTCTGTAGAATCTTCTCGCTGC
ABA3(AS-RT)-R	GGTCAGCAACACTCTCCTCGGA
HRD1B(AS-RT)-F	CTACTCCTTCTGTGACTTTGCTCTC
HRD1B(AS-RT)-R	CTTCCCATTGACCTTCCTTGAG
PAP1(AS-RT)-F	CCTAAGTTTCGAGATCCACATTTC
PAP1(AS-RT)-R	GTAGCGGTAGAAAGGAGAGATGAG
NAC062(AS-RT)-F	ATGAGCAATGATGTTTCAGTGACG
NAC062(AS-RT)-R	AGACTGACATCAAGAACACAACTGC
bZIP63(AS-RT)-F	AGAATCAGCTAGACGGTCCAGAAG
bZIP63(AS-RT)-R	TGTTTGAGTTACATCAGTGAGACCC
CKG(AS-RT)-F	GAAGTAGGAACGTCTTTTACCAATG
CKG(AS-RT)-R	CAGAAGTTGTTGTTGTTGAACCCAC
BBX17(AS-RT)-F	CTACCTAAGCAGGCTCCTTTGTC
BBX17(AS-RT)-R	CACATCATTCGATATCTCGTTTG
FRS1(AS-RT)-F	ATCAAGTTTCAGACCAGAAGCTATG
FRS1(AS-RT)-R	ATTCCTGTTGGAGTGTTGATGTTTC
bHLH23(AS-RT)-F	AGCTCGTGATTCGACGTCTAGTAAG
bHLH23(AS-RT)-R	GTTCTTGCAAAGCCTTCATCATC
PIN4(AS-RT)-F	GTGGCTATGCCCAAAATATTACAAC
PIN4(AS-RT)-R	CAATGGCAATCCCAGCAACAG
ACT2(RT-PCR)-F	GTGATGAAGCACAATCCAAGAGAG
ACT2(RT-PCR)-R	GCCCATCGGGTAATTCATAGTTCT
**RNA FISH probes**	
PIF4-INTRON5-PROBE1	ACAUUUUACCAAAAUCGUCGUUUAAUAAACACGGCUUCGUAUUGGCCUUA
RVE1-INTRON3-PROBE1	GAGGGUCCAGGUAAGGCCAUAACUCAGUAGCAAGAGAUAUUACUAAAUCA
ABA3-INTRON17-PROBE1	UAUUACAAGCAGCUGAUGUAAGACAGCACAUGAUAAGGAGCAAUGCAUAC
HRD1B-INTRON1-PROBE1	AAUAUAGGGCUACAAUGAUAUCUAAACCGAAAGGUCAGUUAUCGUAGACC
