# Supplementary material for: Light regulates nuclear detainment of intron-retained transcripts through COP1-spliceosome to modulate photomorphogenesis
Source: Nat Commun. 2024 Jun 15;15:5130. doi: 10.1038/s41467-024-49571-9 (PMC11180117; doi:10.1038/s41467-024-49571-9)
Supplement: Supplementary file 3 — Description of Additional Supplementary Files [file 41467_2024_49571_MOESM3_ESM.pdf]

Description of Additional Supplementary Files

File Name: Supplementary Data 1

Description: RNA-seq data of alternative splicing events.

File Name: Supplementary Data 2

Description: RNA-seq data of differentially expressed genes.
