# Supplementary material for: Light regulates nuclear detainment of intron-retained transcripts through COP1-spliceosome to modulate photomorphogenesis
Source: Nat Commun. 2024 Jun 15;15:5130. doi: 10.1038/s41467-024-49571-9 (PMC11180117; doi:10.1038/s41467-024-49571-9)
Supplement: Supplementary file 6 — Reporting Summary [file 41467_2024_49571_MOESM6_ESM.pdf]

nature portfolio

Corresponding author(s): Xing Wang Deng

Last updated by author(s): May 24, 2024

Reporting Summary

Nature Portfolio wishes to improve the reproducibility of the work that we publish. This form provides structure for consistency and transparency in reporting. For further information on Nature Portfolio policies, see our Editorial Policies and the Editorial Policy Checklist.

Statistics

For all statistical analyses, confirm that the following items are present in the figure legend, table legend, main text, or Methods section.

n/a	Confirmed	
☐	☒	The exact sample size (*n*) for each experimental group/condition, given as a discrete number and unit of measurement
☐	☒	A statement on whether measurements were taken from distinct samples or whether the same sample was measured repeatedly
☐	☒	The statistical test(s) used AND whether they are one- or two-sided<br>*Only common tests should be described solely by name; describe more complex techniques in the Methods section.*
☒	☐	A description of all covariates tested
☒	☐	A description of any assumptions or corrections, such as tests of normality and adjustment for multiple comparisons
☐	☒	A full description of the statistical parameters including central tendency (e.g. means) or other basic estimates (e.g. regression coefficient) AND variation (e.g. standard deviation) or associated estimates of uncertainty (e.g. confidence intervals)
☐	☒	For null hypothesis testing, the test statistic (e.g. *F*, *t*, *r*) with confidence intervals, effect sizes, degrees of freedom and *P* value noted<br>*Give P values as exact values whenever suitable.*
☒	☐	For Bayesian analysis, information on the choice of priors and Markov chain Monte Carlo settings
☒	☐	For hierarchical and complex designs, identification of the appropriate level for tests and full reporting of outcomes
☒	☐	Estimates of effect sizes (e.g. Cohen's *d*, Pearson's *r*), indicating how they were calculated

Our web collection on statistics for biologists contains articles on many of the points above.

Software and code

Policy information about availability of computer code

Data collection	For real-time PCR: StepOne Software v2.2.3, QuantStudio Real-Time PCR software.<br>For western blot: AllDoc_x (Tanon).<br>For BiFC and RNA FISH: ZEN.
Data analysis	For statistical analyses: Microsoft Excel and GraphPad Prism version 5.0.<br>For phenotype analysis and western blot quantification: Image J.<br>For genome resequencing: LifeScope version 2.5, Perl.<br>For mRNA-seq: StringTie (v1.3.3b), FeatureCounts v1.5.0-p3, DESeq2, Hisat2, StringTie, rMATS (v4.1.2), IGV_2.8.3.<br>For LC-MS/MS analysis: Proteome Discoverer.<br>For phylogenetic construction: InterPro (v89.0), Phyutility (v2.2.6), IQ-TREE (v2.1.4-beta), iTOL, MAFFT (v7.475), HMMER (v3.3.1).<br>For protein structure: PyMOL.

For manuscripts utilizing custom algorithms or software that are central to the research but not yet described in published literature, software must be made available to editors and reviewers. We strongly encourage code deposition in a community repository (e.g. GitHub). See the Nature Portfolio guidelines for submitting code & software for further information.

Data

Policy information about availability of data

All manuscripts must include a data availability statement. This statement should provide the following information, where applicable:

- Accession codes, unique identifiers, or web links for publicly available datasets
- A description of any restrictions on data availability
- For clinical datasets or third party data, please ensure that the statement adheres to our policy

All materials in this study are available from the corresponding author upon request. RNA-seq data have been deposited in the NCBI GEO under accession number PRJNA880452. The mass spectrometry proteomics data have been deposited to the ProteomeXchange Consortium via the PRIDE partner repository with the dataset identifier PXD043190.

Research involving human participants, their data, or biological material

Policy information about studies with human participants or human data. See also policy information about sex, gender (identity/presentation), and sexual orientation and race, ethnicity and racism.

Reporting on sex and gender	n/a
Reporting on race, ethnicity, or other socially relevant groupings	n/a
Population characteristics	n/a
Recruitment	n/a
Ethics oversight	n/a

Note that full information on the approval of the study protocol must also be provided in the manuscript.

Field-specific reporting

Please select the one below that is the best fit for your research. If you are not sure, read the appropriate sections before making your selection.

☒ Life sciences ☐ Behavioural & social sciences ☐ Ecological, evolutionary & environmental sciences

For a reference copy of the document with all sections, see nature.com/documents/nr-reporting-summary-flat.pdf

Life sciences study design

All studies must disclose on these points even when the disclosure is negative.

Sample size	At least 20 seedlings were used for phenotype analysis. No statistical method was used to predetermine sample sizes.
Data exclusions	No data were exclued.
Replication	Experiments were performed three times with similar results.
Randomization	All samples were allocated randomly into experimental groups.
Blinding	The blinding design is not applicable to this system.

Reporting for specific materials, systems and methods

We require information from authors about some types of materials, experimental systems and methods used in many studies. Here, indicate whether each material, system or method listed is relevant to your study. If you are not sure if a list item applies to your research, read the appropriate section before selecting a response.

Materials & experimental systems

n/a	Involved in the study
☐	☒ Antibodies
☒	☐ Eukaryotic cell lines
☒	☐ Palaeontology and archaeology
☒	☐ Animals and other organisms
☒	☐ Clinical data
☒	☐ Dual use research of concern
☐	☒ Plants

Methods

n/a	Involved in the study
☒	☐ ChIP-seq
☒	☐ Flow cytometry
☒	☐ MRI-based neuroimaging

Antibodies

Antibodies used: anti-UGPase (1:2000, Agrisera, AS05086), anti-Histone H3 (1:2000, ABclonal, A2348), anti-COP1 (1:1000, McNellis et al., 1994), anti-GFP (1:2000, Abmart, M20004L) and anti-Actin (1:2000, Sigma-Aldrich, A0480), anti-DCS1, anti-DCS3, anti-DCS4, anti-DCS5 and anti-DCS6 (1:1000) are custom-made antibodies.

Validation: The anti-DCS1 and anti-DCS3 were identified using the overexpression lines and prokaryotically expressed recombinant proteins. The anti-DCS5 and anti-DCS6 were identified using dcs5 or dcs6 mutants as the control.

Dual use research of concern

Policy information about dual use research of concern

Hazards

Could the accidental, deliberate or reckless misuse of agents or technologies generated in the work, or the application of information presented in the manuscript, pose a threat to:

No	Yes
☒	☐ Public health
☒	☐ National security
☒	☐ Crops and/or livestock
☒	☐ Ecosystems
☒	☐ Any other significant area

Experiments of concern

Does the work involve any of these experiments of concern:

No	Yes
☒	☐ Demonstrate how to render a vaccine ineffective
☒	☐ Confer resistance to therapeutically useful antibiotics or antiviral agents
☒	☐ Enhance the virulence of a pathogen or render a nonpathogen virulent
☒	☐ Increase transmissibility of a pathogen
☒	☐ Alter the host range of a pathogen
☒	☐ Enable evasion of diagnostic/detection modalities
☒	☐ Enable the weaponization of a biological agent or toxin
☒	☐ Any other potentially harmful combination of experiments and agents

Plants

Seed stocks	cop1-6, pif4-2, rve1-2 (SAIL_326_A01), aba3-3, dcs1-4 (SALK_116275) and dcs cop1-6 mutans were used in this study.<br>Transgenic lines PIF4-YFP WT, myc-RVE1 WT, YFP-ABA3 WT, PIF4-YFP cop1-6, myc-RVE1 cop1-6, YFP-ABA3 cop1-6, PIF4-YFP myc-RVE1 cop1-6, YFP-ABA3 myc-RVE1 cop1-6, YFP-DCS1 WT, YFP-DCS2 WT, HA-DCS3 WT, flag-DCS4 WT, YFP-DCS5 WT, YFP-gDCS6 WT, myc-DCS1-2 cop1-6, YFP-DCS2-1 cop1-6, myc-DCS2-1 cop1-6,myc-gDCS3-1 cop1-6, DCS4-1 flag cop1-6, YFP-DCS5-1 cop1-6, myc-DCS5-1 cop1-6, and YFP-DCS6-1 cop1-6 were used in this study.
Novel plant genotypes	aba3-3, pif4-2 rve1-2, pif4-2 aba3-3, rve1-2 aba3-3, and pif4-2 rve1-2 aba3-3 mutants all exhibited shortened hypocotyl phenotypes when grown in the dark. PIF4-YFP WT, myc-RVE1 WT, and YFP-ABA3 WT seedling all exhibited elongated hypocotyl phenotype in white light. Moreover, overexpression of PIF4, RVE1, and ABA3 in the cop1-6 background significantly repressed the constitutively photomorphogenic phenotype of cop1-6 in darkness.<br>dcs cop1-6 mutants all displayed cop1-6 suppressor phenotype.
Authentication	cop1-6 was checked by sequencing.<br>aba3-3 mutant, containing a large DNA fragment deletion from C1049 to A2892 of the genomic sequence of ABA3, was created by using the CRISPR/Cas9 technique and checked by sequencing.<br>pif4-2, rve1-2, and dcs1-4 (SALK_116275) were checked using PCR.<br>PIF4-YFP WT, myc-RVE1 WT, YFP-ABA3 WT, YFP-DCS1 WT, YFP-DCS2 WT, HA-DCS3 WT, flag-DCS4 WT, YFP-DCS5 WT, and YFP-gDCS6 WT were confirmed using western blot.
